# Timing of circadian genes in mammalian tissues

**DOI:** 10.1038/srep05782

**Published:** 2014-07-22

**Authors:** Anja Korenčič, Rok Košir, Grigory Bordyugov, Robert Lehmann, Damjana Rozman, Hanspeter Herzel

**Affiliations:** 1 Institute for Theoretical Biology, 10115 Berlin, Germany; 2 Centre for Functional Genomics and Biochips, Institute of Biochemistry, Faculty of Medicine, University of Ljubljana, 1000 Ljubljana, Slovenia; 3 Diagenomi Ltd, 1000 Ljubljana, Slovenia

## Abstract

Circadian clocks are endogenous oscillators driving daily rhythms in physiology. The cell-autonomous clock is governed by an interlocked network of transcriptional feedback loops. Hundreds of clock-controlled genes (CCGs) regulate tissue specific functions. Transcriptome studies reveal that different organs (e.g. liver, heart, adrenal gland) feature substantially varying sets of CCGs with different peak phase distributions. To study the phase variability of CCGs in mammalian peripheral tissues, we develop a core clock model for mouse liver and adrenal gland based on expression profiles and known *cis*-regulatory sites. ‘Modulation factors’ associated with E-boxes, ROR-elements, and D-boxes can explain variable rhythms of CCGs, which is demonstrated for differential regulation of *cytochromes P450* and 12 h harmonics. By varying model parameters we explore how tissue-specific peak phase distributions can be generated. The central role of E-boxes and ROR-elements is confirmed by analysing ChIP-seq data of BMAL1 and REV-ERB transcription factors.

An endogenous circadian timing system controls daily rhythms in physiology, metabolism, and behavior^1^. Autonomous rhythms are generated by intracellular transcriptional feedback loops involving *cis*-regulatory elements such as E-boxes, D-boxes, and ROR-elements (RREs)^2^. Furthermore, up to 10% of all mammalian genes oscillate in their expression levels with a period of about 24 h^3^,^4^. These clock-controlled genes (CCGs) modulate essential physiological processes in an optimized tissue-specific manner. Examples are the gating of cell division *via Myc* and *Wee1*^5^, the control of metabolism due to rhythmic nuclear receptors^6^, the modulation of the immune response^7^, and diurnal rhythms of detoxification *via Alas1* and other factors^8^.

Genome-wide transcription profiles reveal that CCGs differ drastically from tissue to tissue^9^. The corresponding gene sets not only exhibit little overlap (about 10%) but also feature quite different peak phase distributions^10^. Understanding the complexity of tissue-specific circadian expression patterns remains a major challenge. Several mechanisms could be responsible for tissue-specific rhythm generation: (1) variations of the core clock across different tissues, (2) tissue-specific rhythmically expressed transcription factors and co-factors, (3) and systemic cues such as hormone secretion, sympathetic innervation, body temperature, and activity rhythms.

We explore the regulation of CCGs by comparing expression profiles in mouse liver and adrenal glands. For both tissues we analyse our own data for core clock genes from light-dark (LD) cycles and constant darkness (DD). Using carefully normalized expression profiles together with experimentally verified circadian *cis*-regulatory elements we derive for both tissues and both conditions a gene regulatory model of the core clock. The expression of a specific clock gene is described with a differential equation with a production term that depends on the concentrations of core clock components and a decay term. Each production term is a product of “modulation factors” that describe the contributions of E-box-, D-box-, and RRE-regulation.

Our data-driven core clock model is extended to simulate expression profiles of other clock-controlled genes. First we model specific rhythmic genes. We show that variability in amplitude and phase of *cytochromes p450* can be reproduced by our models with variations in the core clock. Then we use our extended model to simulate tissue-specific phase distributions by sampling the parameter space in a biologically plausible range. We compare the range of behavior that could be expected for CCGs with particular regulatory regions to available ChIP-seq and transcriptome data. Much of the phase variability can be traced back to E-box and ROR-element regulations. We list candidates of additional co-regulatory transcription factors. Our modelling approach is particularly valuable to understand the mechanism of 12 h rhythms observed in about 1% of mouse liver genes^4^. We show that multiplicative regulation by core clock components can generate harmonics.

## Results

### Core clock models reproduce expression profiles

In order to compare circadian rhythms in the liver and adrenal gland under different conditions (LD vs. DD), we generated expression profiles of 21 selected clock-related genes based on quantitative time-resolved RT-PCR data (Supplementary Dataset 1, Supplementary Fig. S1). Using a common normalization method *via* three reference genes^11^ we obtained reliable amplitudes and waveforms (Supplementary Table 1). Our data are consistent with previously published expression profiles from genome-wide studies that will be analysed to obtain phase distributions^4^,^12^.

**Figure 1:**
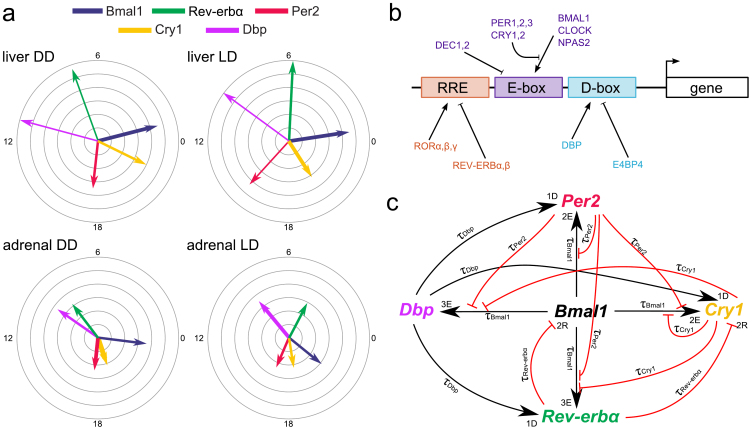
Modelling the core clock. (a) - Measured expression profiles of core clock genes in different tissues and conditions. Arrow direction represents the phase (expressed in the units of circadian time), its length the amplitude, and line thickness the width of the gene's expression peak. See also Supplementary Fig. S1, Supplementary Dataset S1 and Supplementary Table S1. (b) - Transcription of clock genes with the main circadian promoter elements: E-boxes, D-boxes, and ROR elements (RRE). (c) - Wiring diagram of our core clock model. Black and red lines represent activating and repressing action, respectively. Each gene influences other gene's transcription after a certain time delay *τ*. Numbers next to the arrowheads show the number of respective promoter elements in the regulatory region (E - E-box; D - D-box; R - RRE). See also Supplementary Figs. S2 and S4, and Supplementary Dataset S2 and Supplementary Table S2.

Figure 1a shows the experimentally measured gene expression characteristics in a circular representation in units of circadian time (CT). CT refers to the phase of an animal's endogenous rhythm in free-running conditions; CT 0 marks the start of the subjective day, and CT 12 the start of the subjective night. The peak phase relationship is comparable in liver and adrenal gland in DD and LD. The relative amplitudes of liver rhythms are somewhat larger than in the adrenal gland. Furthermore, some genes (e.g. *Bmal1* and *Cry1*) peak earlier in the adrenal gland (Supplementary Fig. 1b). Differences between LD and DD conditions are relatively small.

There are comprehensive mathematical models of the mammalian core clock including the E-box transcription factors (BMAL1, CLOCK, NPAS2), inhibitors of E-box-driven transcription (PER1,2,3: CRY1,2; DEC1,2), nuclear receptors (ROR*α*, *β*, *γ*: REV-ERB*α*, *β*), and D-box-binding transcription factors (DBP, E4BP4, TEF, HLF)^2^,^13^,^14^,^15^. Figure 1b summarizes the current understanding of these circadian promoter elements and their regulators. Our aim is to construct a minimal mathematical model that can represent the core clock in different tissues and conditions. We want to include all three known regulatory loops (with E-boxes, ROR-elements, and D-boxes) while maintaining a manageable number of model parameters with a clear biological meaning.

As a first simplification in our model, we merge poorly characterized intermediate steps (post-translational modifications, complex formation, nuclear localization) into explicit delays of several hours. In this manner, the number of components and kinetic parameters can be reduced drastically. Secondly, we lumped together redundant regulators based on our measured expression data (see Supplementary Methods for details). Our resulting minimal gene regulatory model is presented in Fig. 1c. It is based on our previously published model^16^ with simplified kinetic terms^17^ and a reduced number of genes. The model quantifies positive and negative transcriptional regulations including delays representing protein dynamics. Since the interconnected feedback loops are based on regulatory regions of the 5 genes, the determination of known *cis*-regulatory elements is an essential part of model construction. In Supplementary Methods we list experimental studies supporting our choice of functional E-boxes, ROR-elements, and D-boxes.

Our 5 model equations consist of production terms including activation and repression, and degradation terms. For example, *Bmal1* transcription is governed by *Rev-erbα* at time *t* − *τ_Rev_*_−*erbα*_, where *τ_Rev_*_−*erbα*_ represents the explicit delay between the peaks of *Rev-erbα* mRNA and protein expression. The regulation can be described as: 
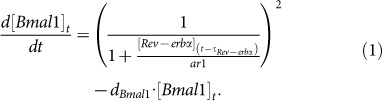
Here, *d_Bmal_*_1_ is the degradation rate of *Bmal1*, and the repression of *Bmal1* by *Rev-erbα* on ROR-elements (RREs) can be described through the parameter *ar*1. The exponent represents the number of functional ROR elements in the regulatory region of *Bmal1*. We introduced the concept of modulation factors that represent transcriptional regulation *via* one circadian regulatory element; in this case only a RRE modulator is used. The complete set of model equations and parameters is given in the Supplementary Methods (Supplementary Eqs. S4–8). Reasonable ranges of degradation rates and delays were taken from large scale studies of mRNA decay^18^,^19^,^20^ and protein measurements^21^,^22^,^23^,^24^ (Supplementary Table 2). As described in Supplementary Methods in detail, we applied evolutionary optimization strategies to estimate unknown parameters. We defined initial values and ranges for all model parameters and evaluated each parameter combination through the computation of a custom score containing constraints for target period length, amplitudes, phases, and peak widths of all genes. The proces was repeated multiple times, each time with narrower ranges for possible parameter values. It turns out that the resulting models can reproduce the period, phases, amplitudes and waveforms according to our defined tolerance ranges (Supplementary Fig. 2).

The successful modelling of expression profiles with our relatively simple 5-gene model suggests that the core clock in mammalian tissues is indeed governed by an interplay of positive and negative feedback loops as postulated by Ueda et al. based on an *in vitro* cell culture system^2^. Since the comparison of expression profiles illustrated in Fig. 1a shows clear similarities such as the order of phases, we also fitted a ‘consensus model’ to all four data sets. This model can be exploited to study generic properties of the regulatory network such as sensitivity to parameter variations.

Control analysis of the consensus model revealed that certain parameters are particularly important (see Supplementary Methods, Supplementary Dataset S2, and Supplementary Figs. S2c and S4). Among these essential parameters are delays (*τ_Bmal_*_1_, *τ_Rev_*_−*erbα*_, *τ_Per_*_2_, *τ_Cry_*_1_), degradation parameters (*d_Rev_*_−*erbα*_, *d_Cry_*_1_), and parameters quantifying transcription *via* E-boxes and RREs (*gr*3, *b_Rev_*_−*erbα*_, *b_Per_*_2_, *b_Cry_*_1_). These findings are in agreement with experimental observations showing that PER2 stability and nuclear localization control period and phases^25^. Interestingly, control analysis indicates that both loops (PER/CRY as well as REV-ERB repression) have significant effects on phase dynamics. This finding confirms an earlier claim that both loops might have comparable relevance for the core clock^15^.

Figure 1a and similarities between the models regarding the parameter values and model behavior indicate that the core clock is robust in different tissues under LD and DD conditions. In contrast, output genes exhibit distinctly variable rhythms. Sets of clock controlled genes in different tissues have small overlaps and different phase distributions. Below, we take our core clock model as a driver of output genes and show that even minor variations in E-box, D-box, and RRE regulation can induce quite large variability of clock-controlled genes.

### Modelling the variability of clock-controlled genes

**Figure 2:**
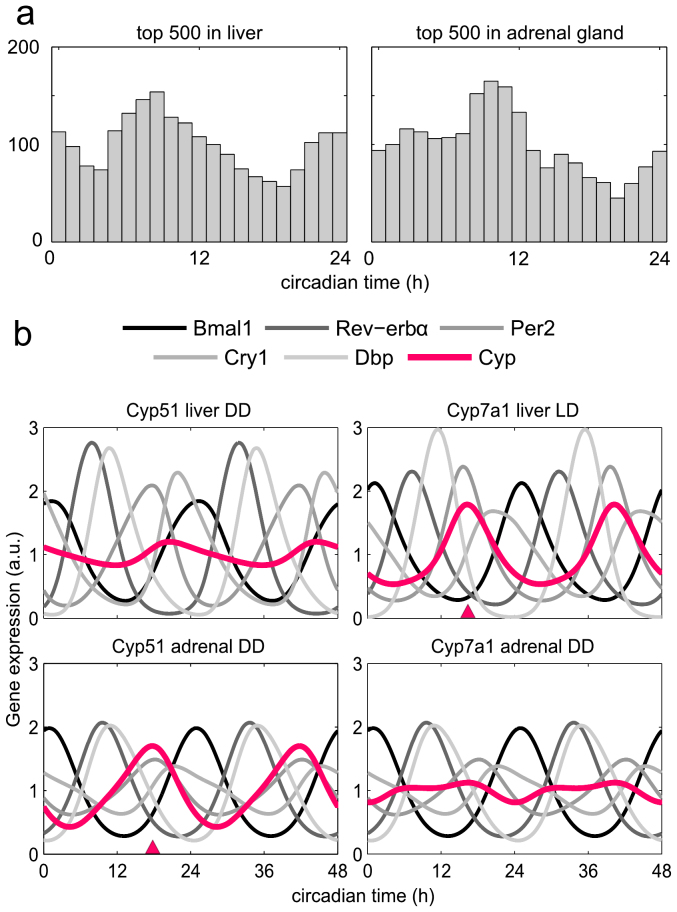
Expression patterns of CCGs. (a) - Phase distributions of rhythmic genes in the liver and adrenal gland. The top 500 probe sets (by amplitude) are shown for each tissue, and the overlap between the sets is only 68 probe sets. Histogram is shown for overlapping 4 h bins. (b) - *Cyp51* and *Cyp7a1* - simulations can describe the observed behaviour. Red triangles represent the phase observed in the experimental data in case of rhythmic expression, and the red line shows the simulated time-course. The gray curves show the 5 simulated core clock genes.

Above we represented the core clock by a gene regulatory model that can describe tissue- and condition-specific expression profiles. In this section we explore the astonishing variability in phases of circadian output genes. We identified clock-controlled genes from publicly available expression data^4^,^12^ by harmonic regression (see Experimental procedures for details). We analyze only genes with above-threshold expression levels and, furthermore, we focus on CCGs with large amplitudes. Figure 2a shows the phase distributions of the top 500 probe sets (by amplitude) in the liver and adrenal gland. Despite the similarity of the core clock rhythms in both tissues (Fig. 1a), we surprisingly find only 68 overlapping probe sets with clearly different peak phase distributions.

We show in this section that minor variations of E-box, D-box and RRE regulation can generate quite variable outputs. As an example we discuss the differential circadian regulation of *cytochrome p450* genes essential for cholesterol and bile acid synthesis. Our qPCR measurements show that *Cyp51* is rhythmic in the adrenal gland but not classified as rhythmic in the liver due to the low amplitude. The opposite is found for *Cyp7a1* (see Supplementary Methods). Reassuringly, our measurements are consistent not only with published microarray studies^4^,^12^ but also with the reported roles of the two enzymes. CYP51 (lanosterol 14*α*-demethylase) catalyzes demethylation of lanosterol in the cholesterol biosynthesis pathway. Most of cholesterol production occurs in the liver and a significant amount is synthesized also in the adrenal gland, where *Cyp51* was found to be circadian^26^. CYP7A1 (cholesterol 7*α*-hydroxylase) is the rate-limiting enzyme in the synthesis of bile acid from cholesterol in the liver. *Cyp7a1* is rhythmically expressed in the liver but not in the adrenal gland.

Figure 2b shows the successful modelling of *cytochrome p450* genes. The red triangles indicate the experimentally measured peak phases. Our models reproduce the phases and the large amplitudes of *Cyp51* in the adrenal gland under DD conditions and of *Cyp7a1* in the liver under LD where its oscillations were most pronounced. The gray and black curves represent the simulated core clock gene rhythms. Minor variations of the core clock oscillations can explain large variations of output genes (see Supplementary Methods - Phase determination of CCGs for details). For example, the effect of *Cry1* modulator is quite pronounced in liver LD leading to large *Cyp7a1* amplitudes. Overall, variability of promoters of clock-controlled genes can tune the relative roles of E-boxes, D-boxes, and RREs.

### Simulations reproduce complex phase distributions

Fig. 2a has shown that the distributions of peak phases of clock-controlled genes are quite diverse. Our examples of *cytochrome p450* genes above illustrate the phase determination of individual CCGs. In the following we exploit our concept of transcriptional modulators to understand phase distributions of output genes.

Just like the core clock genes, clock-controlled genes are also driven by the same regulators and can thus be represented by the same modelling framework with E-box-, D-box, and RRE modulators. First we studied a hypothetical output gene *CCG* driven solely by a ROR-element. The expression of such a gene is given by the following equation with an RRE modulator as production term and a decay term: 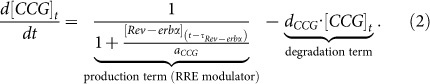
Typical mRNA degradation rates *d_CCG_* are 0.2–0.6 *h*^−1^ 18,19,20. The repression *via* Rev-erb*α* is modelled by the denominator of the production term. The time-course of Rev-erb*α* is given by the core clock model described above. The remaining parameter *a_CCG_* depends on the specific characteristics of the CCG promoter and can be affected by the position of the ROR element, co-factors, and chromatin architecture^27^. Consequently, the parameters *a_CCG_* and *d_CCG_* vary from gene to gene^28^.

**Figure 3:**
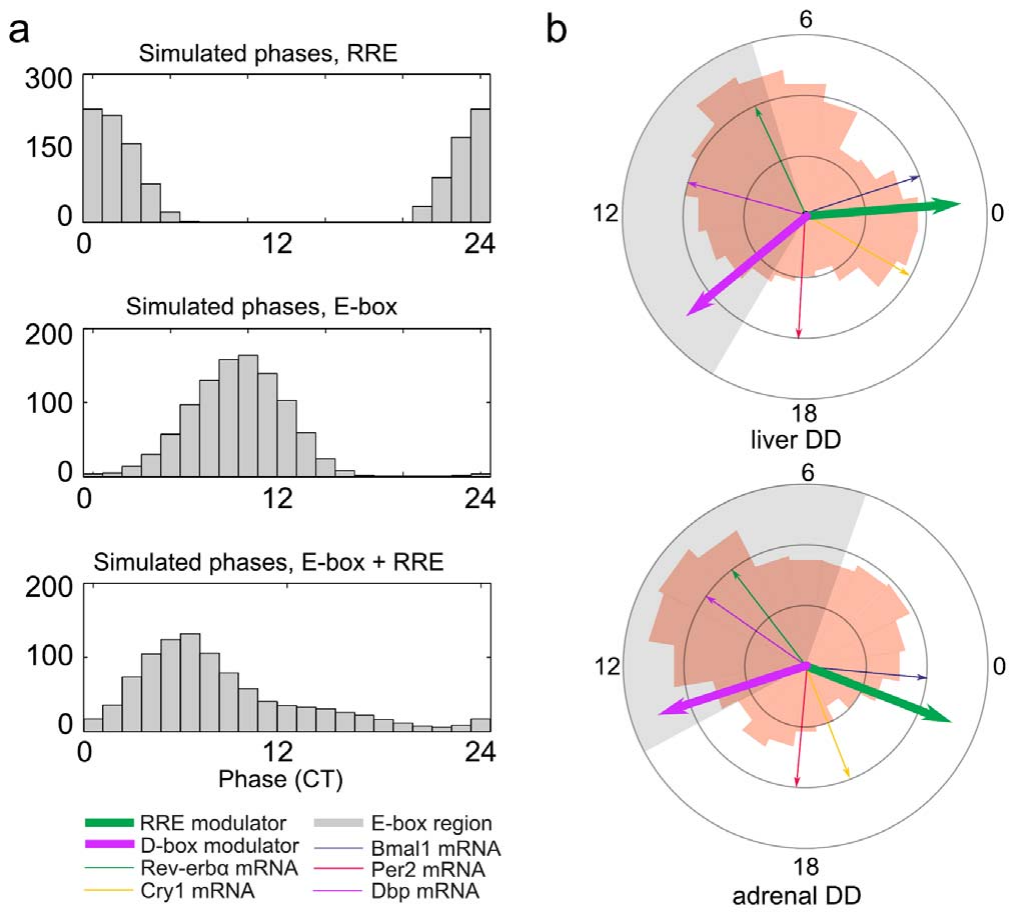
E-box, D-box, and RRE modulators govern entrainment phases. (a) - Simulated phase distributions of clock output genes governed by RREs, E-boxes, and both (Supplementary Eq. 9). (b) - Phases of modulation factors. Green and violet arrows show the peak phases of RRE and D-box modulation factors. The orange phase distribution shows a phase distribution of top 500 circadian probe sets in the liver and adrenal gland. The phase is not determined by a single regulator but by a combination of transcriptional modulators. The range of phases of E-box modulators is represented by the shaded area; if driven mostly by *Bmal1*, the phase would be around CT 6, or later if driven by *Cry* and *Per* derepression. See also Supplementary Fig. S3 for simulated peak phase distribution of genes governed by D-boxes and Supplementary Fig. S5.

In Fig. 3a we chose *a_CCG_*, *d_CCG_*, and *τ_Rev_*_−*erbα*_ randomly within the range observed in core clock models. Simulation of 250 output genes using these parameter ranges yields the peak phase distribution shown in Fig. 3a (top graph). There is a pronounced peak around CT 0 for RRE-driven clock-controlled genes. This timing is the result of a series of steps: Rev-erb*α* is an immediate early target of BMAL1:CLOCK and has a sharp expression peak near CT 6 (Fig. 1a). The inhibition of target genes by REV-ERB*α* is delayed according to our model by 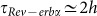. This implies that repression is released at opposite phase - around CT 20. Variable half-lives of individual RRE-driven genes lead to a further delay of their expression peaks by a few hours (Supplementary Methods and Supplementary Fig. S5). Taken together, these regulatory principles (delayed inhibition, gene-specific half-lives) explain the clustering of peak phases of RRE targets around CT 0.

Generalizing Equation (2), the production term of a CCG can be a product of additional modulation factors describing regulation through D-boxes and E-boxes based on the regulatory regions of specific CCGs. The D-box modulator describes regulation of transcription through *Dbp* that has its expression peak around CT 11 and induces genes after a delay of about 2 h. Consequently, many D-box targets exhibit maximal expression levels around CT 15 (Supplementary Fig. 3a).

The concept of ‘E-box modulators’ is somewhat more complicated since we have a concerted action of multiple regulators. BMAL1 serves as an activator whereas PER and CRY proteins repress E-box driven transcription. Consequently, transcriptional regulation is achieved by a balance of activation and repression modelled by products of regulatory terms (see Supplementary Eqs. S4–8 in Supplementary Methods). ChIP-seq data indicate that BMAL1 binds to E-boxes around CT 6^24^,^29^. PER and CRY proteins bind between CT 15 and CT 4 and suppress transcription *via* E-boxes. Thus E-box target genes are typically transcribed between CT 6 and CT 14. Since our model includes the activator *Bmal1*, the early repressor *Per2*, and the late repressor *Cry1*, much of the complexity of E-box regulation can be simulated. Fig. 3a (middle graph) shows that simulated target genes exhibit the expected phase distribution with a peak centered around CT 10.

As discussed above, the concept of E-box-, D-box-, and RRE-modulators is helpful to understand phase distributions of clock-controlled genes. Modulators combine the effects of multiple transcriptional regulators and allow the prediction of output phases based on core clock data. Gene-specific variabilities of parameters such as *a_CCG_*, *d_CCG_*, and *τ_Rev_*_−*erbα*_ in Equation (2) lead to distribution of simulated output phases as illustrated in Fig. 3a. The lowest panel of Fig. 3a shows an example where both E-boxes and RREs govern the phase. In this case, we simulate intermediate phases between E-box- and RRE-driven transcription.

Now we compare simulated peak phases of our modulators with experimental phase distributions of clock-controlled genes in the liver and adrenal gland. The circular diagrams in Fig. 3b show that the variability of output phases can be associated with E-box-, D-box-, and RRE-modulators to a certain extent. Thick arrows show the peak activity of D-box- and RRE-modulators. The shaded area shows the activity phases of the E-box-modulator: if driven mostly by activation through *Bmal1*, the phase would be around CT 6. If the phase is governed by the derepression by *Cry* and *Per*, later phases are possible.

The orange phase distribution in Fig. 3b shows a phase distribution of top 500 circadian probe sets from Fig. 2a in the liver and adrenal gland. According to our model, RRE targets are expected to peak around CT 0. The corresponding peak in liver is more pronounced which could indicate stronger regulatory effects of Ror*γ* and Rev-erb*α*. Indeed, ChIP-seq experiments found more than 20.000 genes bound by REV-ERBs in liver^30^. Simulations show that genes dominated by E-boxes and D-boxes are expected to have expression peaks around CT 5–12 (E-boxes, Fig. 3a) and CT 14 (D-boxes, Supplementary Fig. S3). We find many CCGs with expression phases in these ranges, especially in the adrenal gland. Comparison of experimental and simulated peak phase distributions (Supplementary Fig. S3) reveals that the differences between the liver and adrenal gland cannot be attributed solely to differences of modulators considered in our model. Consequently, we discuss below additional tissue-specific regulators.

### Comparison with ChIP-seq data

As discussed above, our core clock model allows us to simulate phase distributions of E-box and RRE target genes (with regulators BMAL1 and REV-ERB*α*). Fortunately, mouse liver data from ChIP-seq experiments are available to test our predictions. Rey et al.^24^ quantified the binding of BMAL1 at 6 different time points and identified about 2000 target genes. Furthermore, REV-ERB*α* and REV-ERB*β* binding was studied at 2 time points leading to more than 20.000 putative REV-ERB target genes^30^,^31^,^32^. For the comparison with our simulations we prepared 3 gene sets: BMAL1 targets excluding REV-ERB targets, REV-ERB targets excluding BMAL1 targets, and common targets of BMAL1 and REV-ERB. The phases of these genes were determined from high-resolution data of^4^.

**Figure 4:**
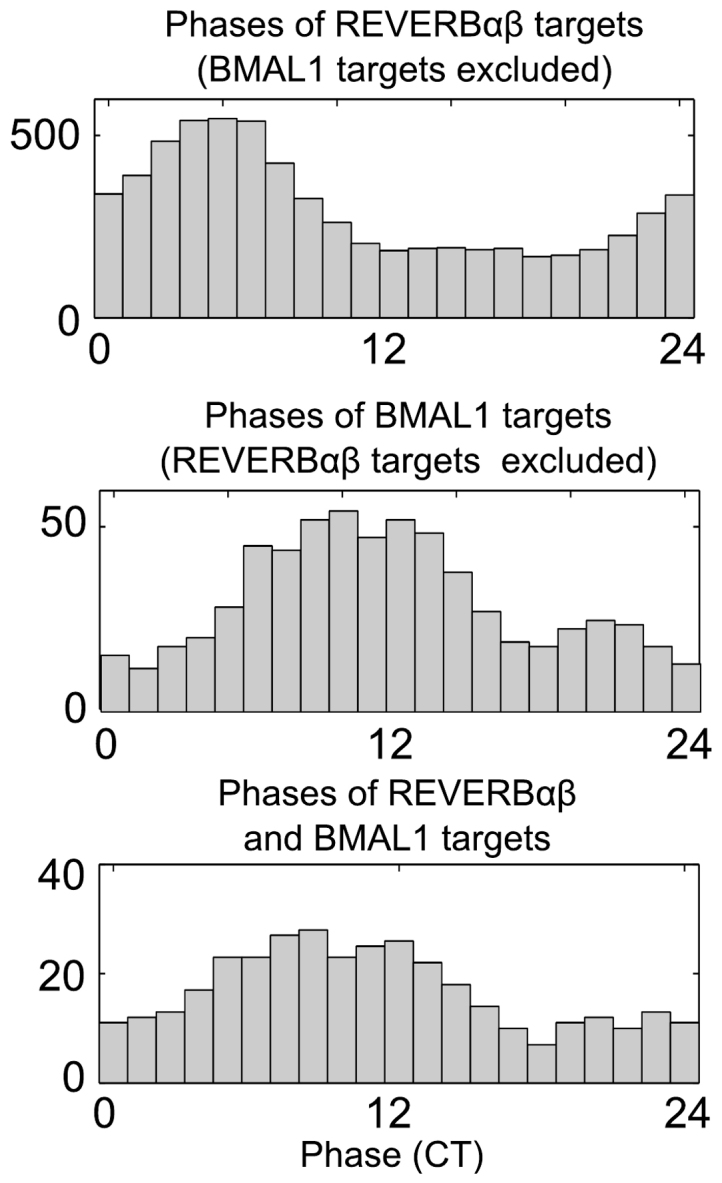
Observed phase distributions from ChIP-seq experiments. BMAL1 and REV-ERB*α*/*β* targets were taken from^24^,^29^ and their phases from^4^.

Figure 4 shows measured phase distributions. As expected, E-box targets have peak expression phases around CT 10 (middle panel). The observed distribution is somewhat broader than the simulated distribution in Fig. 3a and also has many genes at late phases around CT 20. We conclude that E-box driven transcription is a major determinant of phases as shown recently also by^29^. However, other factors such as D-box regulators, HSF, SRF, and CEBP can influence expression phases as well^33^,^34^,^35^. Furthermore, periodic degradation induced, e.g., by periodic polyadenylation^36^ and circadian control of ribosome biogenesis^37^ can lead to additional peaks of expression phases.

Figure 4 (top panel) represents the REV-ERB targets excluding E-box targets. Here our simulations (Fig. 3a, top panel) predict a relatively sharp peak after CT 0. The measured experimental distribution is somewhat broader and has its maximum at CT 5. These differences suggest that other transcriptional or post-transcriptional regulators might contribute to the phase determination of REV-ERB targets.

Simulations also allow us to generate peak phase distributions of genes with both regulatory regions, E-boxes and RREs. Our simulated phase distribution has a broad asymmetric peak early in the day (Fig. 3a, lower panel). The experimental counterpart, genes with measured BMAL1 and REV-ERB binding (Fig. 4, lower panel), exhibits a very broad phase distribution centered at CT 8. This observation suggests that the combinatorial action of two regulators allows quite variable phases. This aspect is further discussed in the next section using model simulations of combinatorial gene regulation. In addition to the regulation by the core clock genes, other transcription factors could be driving gene expression at different phases. Table 1 lists selected candidate transcription factors. Some of them are expressed in both tissues (with partly different phases), and others are specific for one tissue only. These rhythmic transcription factors are candidates for an additional level of phase regulation in output genes.

### Combinatorial gene regulation broadens phase distributions and generates harmonics

Most genes are regulated by multiple transcription factors. If several factors bind to the same regulatory region (e.g. DBP and E4BP4), their overall effect can be modelled by a common modulation factor as explained in Supplementary Methods. Thermodynamic considerations^17^ show that binding to distinct boxes can be modelled by products of modulation factors^38^.

Our 5-gene model contains several products of regulatory terms (see Supplementary Eq. 4–7). Here we demonstrate the effects of combinatorial regulation by a simplified product of two phase-shifted modulators, i.e., we study the multiplication of sinusoidal terms: 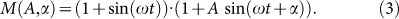
The second sine-term has an amplitude *A* ≤ 1 and is phase-shifted by *α*. Multiplication and trigonometric identities lead to: 
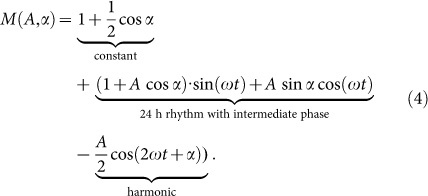
As discussed earlier by Ueda^2^,^39^, the combined action of two regulators leads to intermediate phases of the transcriptional production term. For small *A* the sine-function dominates whereas for large *A* intermediate phases are possible. The geometrical interpretation was termed ‘phase vector model’^39^. Interestingly, for *A* close to 1 and *α* = 180° (out-of-phase activators) the sine vanishes leading to harmonic oscillations with the double frequency. For circadian periods this corresponds to 12 h rhythms. Such harmonics were found in about 1% of mouse liver genes^4^.

**Figure 5:**
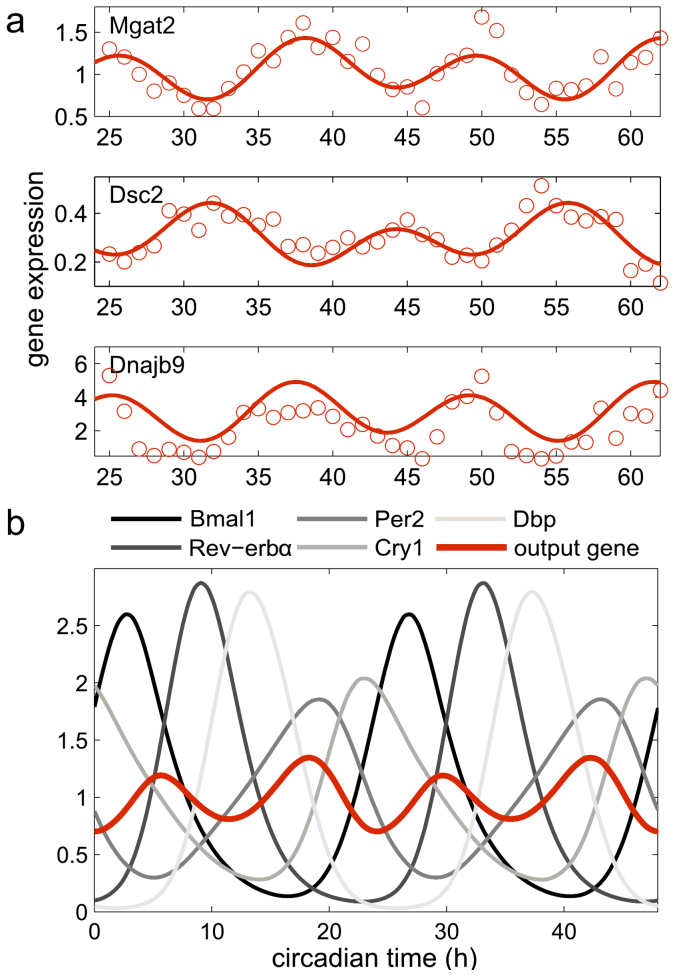
Harmonics in circadian gene expression. (a) - Examples of high-amplitude 12 h rhythmic gene expression as measured by^4^. (b) - Simulated gene expression with a 12 h period taking into account regulation through E-boxes, ROR-elements, and D-boxes (Supplementary Eq. 9).

In our model we have products of 3 modulation factors of quite different phases. Consequently, harmonics are possible for appropriate parameter values. Figure 5 shows experimentally measured 12 h rhythms observed by^4^ and simulations of our liver model. Even without considering additional rhythmically transcribed factors as studied in^38^, the multiplicative action of D-boxes, E-boxes, and RREs allows the generation of 12 h transcription profiles. Our model is thus a useful tool to study and analyze clock-related phenomena including generation of harmonics.

## Discussion

**Figure 6:**
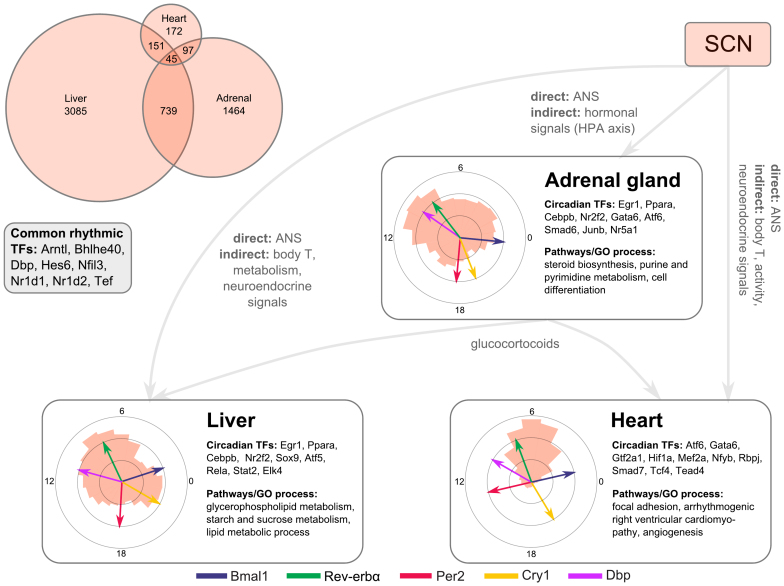
Local and systemic regulation of CCGs. Upper left: Small overlap of CCGs in different tissues but common rhythms of 7 core clock genes. Inserts: Rhythmically expressed transcription factors and overrepresented features in CCG sets in the liver, adrenal gland and heart. See also Supplementary Dataset S3.

Expression profiling has shown that sets of clock-controlled genes in different peripheral tissues have surprisingly little overlap. Furthermore, peak phases of CCGs differ drastically (Fig. 6). These tissue-specific features might result from variations of the core clock, from tissue-specific co-regulators, or from different systemic inputs.

Based on carefully normalized qPCR data and known regulatory interactions we derived a core clock model for liver and adrenal gland under DD and LD conditions. Degradation rates and delays were largely adopted from literature values whereas unknown parameters of transcriptional regulation were derived by evolutionary optimization techniques. Even though the resulting core clock models were relatively similar, variations in amplitude and phases allowed us to reproduce striking differences of *cytochrome p450* gene rhythms (see Fig. 2).

Our gene regulatory models include transcriptional regulation terms (based on known *cis*-regulatory elements) and degradation terms. We introduce ‘modulation factors’ associated with regulations *via* E-boxes, D-boxes, and ROR-elements. The phases of these modulation factors depend on the delays between gene expression and regulatory action and on the balance between activation and repression. Modulation factors allow direct predictions of phases of clock-controlled genes (see Fig. 3). Simulations show that RRE-modulators lead to phase distributions centered around CT 0, whereas E-box modulators allow a broad range of phases during the subjective day. These predictions are largely consistent with phase distributions derived from ChIP-seq data and genome-wide expression profiles (compare Fig. 3a and Fig. 4).

There are, however, still clear differences between core-clock based predictions and observed phase distributions as shown in Fig. 3 and 4. Consequently, we explored putative transcriptional co-regulators. In Table 1 and Fig. 6, rhythmically expressed transcription factors are listed that may be involved in fine-tuning of phases. Below we explore the associated pathways in some detail.

High-resolution expression profiling revealed that about 1% of liver genes display 12 h rhythms termed harmonics^4^. Our concept of modulation factors provides a straight-forward mechanism to obtain harmonics *via* multiplicative combinatorial regulation: out-ouf-phase activators may annihilate 24 h rhythms but can also generate 12 h rhythms. Recently, candidates of such out-of-phase activators were compiled^38^. Our model shows that also core-clock regulators themselves can generate harmonics (see Fig. 5). Simulations demonstrate the interplay of core-clock elements in regulation of rhythmically expressed genes including genes with 12 h periodicity.

Above we addressed the question how the observed tissue-specific regulation of circadian rhythms is achieved. Using models of the core clock we could explain tissue-specificity partially: differential regulation of *cytochrome p450* genes was reproduced, expression phases around CT 0 were traced back to RRE elements, and harmonics could be generated by out-of-phase activators without the need for additional regulators outside the core clock.

However, there are many observations that cannot be explained by core clock variability alone. Comparisons of peak phase distributions from different tissues (Supplementary Fig. S7) show that the peak phase distribution of circadian genes in the heart is strikingly different from other tissues. Most of the circadian genes in heart exhibit expression peaks around CT 6 (see also Fig. 6) even though peak phases of core clock genes (coloured arrows) are similar to other tissues. Consequently, we discuss in the following regulatory mechanisms beyond core clock modulators that could lead to such tissue-specific differences.

Obviously, rhythmic transcription factors can tune the phases of CCGs. We compiled a list of circadian transcription factors in different tissues (see Fig. 6). Among the 8 transcription factors commonly found in the liver, adrenal gland, and heart we find 7 core clock regulators and *Hes6*, which has been reported earlier in a circadian context^40^.

In Table 1 and Supplementary Dataset S3 we list circadian transcription factors sorted according to their amplitudes based on published expression profiles^4^,^12^. Many of their binding motifs have been reported to be overrepresented in CCG promoters^9^,^41^ including EGR, PPAR, CEBP, STAT, and HES. Interestingly, some of these transcription factors (EGR, CEBP, STAT5a) have quite different expression phases in liver and adrenal gland (Table 1).

In order to connect tissue-specific CCGs and rhythmic transcription factors to physiological functions, we performed a Gene Ontology (GO) analysis of clock genes (Supplementary Dataset 6) and looked for overrepresented biological processes and pathways *via* DAVID^42^. As expected, the term ‘circadian rhythms’ scores highly in all 3 tissues. Among the top hits we find glycerophospholipid, starch, and sucrose metabolism in the liver, and steroid biosynthesis in the adrenal gland. In the heart, focal adhesion and arrhythmogenic right ventricular cardiomyopathy are overrepresented.

These processes can be related to rhythmic transcription factors displayed in Fig. 6. *Egr1* is involved in response to glucose and insulin stimuli^43^ and might drive many CCGs in the liver. *Nr5a1* plays a role in adrenal gland development and hormone metabolism^44^. In the heart, *Mef2a* regulates glucose metabolism^45^, and *Gata6* is relevant for heart development^46^. Interestingly, *Atf* transcripts oscillate in all 3 tissues and are known as mediators of cellular stress^47^.

Tissue specificity can be achieved also by different systemic inputs. As illustrated in Fig. 6, peripheral organs receive signals from the autonomous nervous system (ANS) and are affected by oscillating body temperature. The adrenal gland is part of the HPA axis and influences other tissues *via* glucocorticoids. A list of glucocorticoid-dependent circadian genes has been provided by^48^. Adrenalectomy changes expression profiles of many cycling metabolic genes in the liver including *Gsk* and *Cyp7a1*.

It is evident that a comprehensive understanding of tissue-specific gene regulation needs a further integration of high-throughput data including nascent RNA profiles^49^, polyadenylation^36^, degradation rates^50^ and circadian proteomes^51^,^52^. Our analysis demonstrated that data-driven mathematical models can serve as a link between self-sustained core clock oscillations and tissue-specific expression profiles.

## Methods

### Ethics statements

All experiments were performed in accordance with relevant guidelines and regulations; the experiments were approved by the Veterinary Administration of the Republic of Slovenia (license numbers 34401-38/2009/2 and 34401-44/2009/2) and were conducted in agreement with the European Convention for the protection of vertebrate animals used for experimental and other scientific purposes (ETS 123), as well as in agreement with the National Institute of Health guidelines for work with laboratory animals.

### Animals and tissue isolation

C57BL/6JOlaHsd mice were kept under a 12 h:12 h LD cycle (lights on at 07:00; lights off at 19:00, free access to food (Harland Tekland 2916) and water) for 3 weeks to achieve entrainment. 60 mice were then sacrificed by cervical dislocation under same LD conditions. 61 mice were transferred into constant darkness for 36 h and were sacrificed under dim red light. In both cases, samples were taken every 2 h (minimum of 4 mice per time point) over a 24 h period. Immediately after sacrifice, liver and adrenal glands were excised, snap frozen in liquid nitrogen and stored at −80°C.

### RNA extraction and cDNA preparation

The liver samples were homogenized, and the total RNA was isolated according to manufacturer instructions (QuickGene RNA tissue kit S, QuickGene 810, FujiFilm LifeScience). Total RNA from one adrenal gland *per* animal was isolated using 500 μl of TRI reagent (Sigma) according to manufactures instructions. The RNA quantity and quality were assessed with NanoDrop and Agilent 2100 Bioanalyzer instruments. DNAse treatment was performed on all of the samples using DNAse I (Roche Applied Bioscience) according to the manufacturer instructions.

The cDNA synthesis was carried out using SuperScript III reverse transcriptase (Invitrogen). Liver RNA (3 μg) was mixed with 20 μl reverse transcriptase master mix, which contained 8 μl 5× first strand buffer, 2 μl 100 mM dithiothreitol, 2 μl 10 mM dNTP mix, 1 μl random primers (Promega 500 ng/ml), 0.75 μl SuperScript III (200 U/ml), 0.75 μl RNAse OUT (Invitrogen), and 5.5 μl RNAse free water, giving a final volume of 40 μl. The reaction mixtures were incubated at 25°C for 5 min, 50°C for 60 min, and 70°C for 10 min. For the adrenal gland, 1 μg of adrenal gland RNA was mixed together with 10 μl of reverse transcriptase master mix which contained 5 μl of 5× first strand buffer, 1.25 μl of 100 mM DTT, 1.25 μl of 10 mM dNTP mix, 0.65 μl of random primers (Promega 500 ng/μl), 0.5 μl of SuperScript III (200 U/μl), 0.5 μl of RNAse OUT (Invitrogen) and 0.85 μl of RNAse free water in a final volume of 25 μl. The reaction mixtures were incubated at 25°C for 5 minutes, 50°C for 60 minutes and 70°C for 10 minutes.

### qPCR

Intron-spanning primers for the clock and reference genes were designed based on the available gene sequences (Supplementary Dataset 1). The primer specificities and amplification efficiencies were validated empirically with melting curve and standard curve analysis of a six-fold dilution series.

Real-time quantitative PCR was performed in a 384-well format on a LightCycler 480 (Roche Applied Science), using SYBR Green I Master (Roche Applied Science). The PCR reactions consisted of 2.5 μl SYBR Green I Master, 1.15 μl RNAse free water, 0.6 μl 300 nM primer mix and 0.75 μl cDNA, to a total volume of 5 μl. Three technical replicates were performed for each sample. The cycling conditions were: 10 min at 95°C, followed by 40 rounds of 10 s at 95°C, 20 s at 60°C, and 20 s at 72°C. The melting curve analyses for determining the dissociation of the PCR products were performed from 65°C to 95°C.

The Cp values of the expressed genes were transformed into quantities by taking into account the primer efficiencies. These quantities were then normalized by a normalization factor, i.e. the geometric mean of the expression of the reference genes *Hmbs*, *Eif2A*, and *Ppib*^11^.

### Model fitting and simulation

Delay-differential equations with constant delays were implemented in MATLAB and solved using the dde23 function. Bifurcation analysis was performed using DDE-BIFTOOL v2.03 implemented in MATLAB.

The values of degradation rates and explicit delays are based on published data and the exponents representing the numbers of experimentally verified binding sites are fixed. The remaining parameter values were fitted by evolutionary optimization strategies to reproduce phases, amplitudes, and waveforms of our measured expression profiles. For each parameter, an initial value and a range in which the parameter space will be randomly sampled was chosen. For each parameter value combination, the system was evaluated through the computation of a custom score value containing constraints for target period length, amplitudes, phases and peak widths of all genes. The process was repeated 10 times (each time with narrower ranges for each parameter value) to achieve fitting errors below experimental variability of the data. Detailed analysis of the process is described in Supplementary Methods. Extension of the model enables simulations of clock output genes (detailed description is provided in the Supplementary Methods). All scripts are available upon request.

### ChIP-seq data analysis

We reanalysed the ChIP-seq data for BMAL1 and REV-ERB*α*/*β* binding^24^,^30^. Whereas BMAL1 peak locations reported in^24^ were obtained from the paper's Supplementary Information S2, the REV-ERB*α*/*β* peak locations reported in^30^ where taken from the Gene Expression Omnibus data sets GSM840528 and GSM840529, respectively. In correspondence with both ChIP-seq experiments, we used the mouse genome build mm9 and obtained the gene annotations *via* the R package BSgenome.Mmusculus.UCSC.mm9. Each peak was then assigned to all genes for which the distance between start codon and peak was smaller than 5 kb. Hereby, the distance considered the peak center as well as the initial base of the start codon. Using this distance, the peak score measures reported for each peak data set, and the parameter d0 = 500, the transcription factor association score (TFAS) was calculated according to^53^. Two additional measures were added: the number of different genes associated to each peak (peak2gene.mapped.count), and conversely the number of different peaks associated to each gene (gene2peak.mapped.count). The lists can be filtered by distance, TFAS, gene2peak.mapped.count, peak2gene.mapped.count. A cutoff of TFAS > 10^24^ and TFAS > 0.5^30^ was then used to obtain BMAL1 and REV-ERB*α*/*β* target genes.

### Microarray and qPCR data analysis

To equalize the differences arising from different analysis methods in each study, we re-analysed the data with our fitting procedure. For gene expression data in liver, the data set from^4^ was considered. We re-analysed their data with our biharmonic fit function; genes with p-value < 0.01 (F-test) were considered to be rhythmically expressed. Additionally, we added cutoffs for expression level (maximum expression level > 600) and amplitudes (relative amplitude > 0.3). For the adrenal gland, we used published gene expression data from^12^. In this case, we used cutoff for expression levels at 500; otherwise, analysis was the same as for the liver gene expression data. For the heart, data from^10^ were used. qPCR data were fitted by the same biharmonic fit function to obtain amplitudes and phases.

### Peak phase distributions

We took the lists from the ChIP-seq analyses (BMAL1 and REV-ERB*α*/*β* targets) and looked if they express circadian behaviour in the gene expression data sets^4^,^12^. For comparison with simulation results, we created 3 lists: BMAL1 only targets, REV-ERB*α*/*β* only targets, and targets of both BMAL1 and REV-ERB*α*/*β*. Phase distributions are plotted as histograms or circular histograms with overlapping bins (4 h bins with 3 h overlap).

### Circadian transcription factors

Based on the work of^38^ we checked for circadianly expressed transcription factors in the liver, adrenal gland adn heart. We started with a curated list of 340 transcription factors from SwissRegulon that are annotated with their target DNA sequence motifs. The list from Swiss Regulon was modified as described in^38^ and the expression profiles were taken from^4^,^10^,^12^.

## Supplementary Material

Supplementary Information Supplementary Information

Supplementary Information Supplementary Dataset 1

Supplementary Information Supplementary Dataset 2

Supplementary Information Supplementary Dataset 3

Supplementary Information Supplementary Dataset 4

## Figures and Tables

**Table 1:** Rhythmically expressed transcription factors in adrenal gland and liver

Circadian only in adrenal gland	Circadian only in liver
*Gata6*	*Ewsr1*
*Atf6*	*Sox9*
*Smad6*	*Sox5*
*Hoxa5*	*Atf5*
*Runx2*	*Rela*
*Junb*	*Stat2*
*Jun*	*Gabpb1*
*Gata2*	*Smarca5*
*Ets2*	*Stat6*
*Nr5a1*	*Ctcf*
*Smad5*	*Bach2*
*Foxc1*	*Elk4*
*Smarca1*	*Mafb*

**TF name**	**Phase in adrenal gland (CT)**	**Phase in liver (CT)**
*Egr1*	10.8	2.4
*Nr2f2*	9.9	5.8
*Ppara*	10.7	8.9
*Cebpb*	3.0	12.1
*Stat5a*	10.0	0.5
*Hes6*	7.9	17.1
*Esr1*	10.5	18.9

Above - Transcription factors that are rhythmic only in one tissue according to^4^,^12^. Below - Selected transcription factors that are rhythmically expressed in both tissues but with different peak expression phases. Phases are estimated from^4^,^12^. See also Supplementary Dataset S3.

Above - Transcription factors that are rhythmic only in one tissue according to^4^,^12^. Below - Selected transcription factors that are rhythmically expressed in both tissues but with different peak expression phases. Phases are estimated from^4^,^12^. See also Supplementary Dataset S3.

## References

[b1] Bass J. & Takahashi J. S. Circadian integration of metabolism and energetics. Science 330, 1349–1354 (2010). 21127246 10.1126/science.1195027 PMC3756146

[b2] Ueda H. R. *et al.* System-level identication of transcriptional circuits underlying mammalian circadian clocks. Nat Genet 37, 187–192 (2005). 15665827 10.1038/ng1504

[b3] Panda S. *et al.* Coordinated transcription of key pathways in the mouse by the circadian clock. Cell 109, 307–320 (2002). 12015981 10.1016/s0092-8674(02)00722-5

[b4] Hughes M. E. *et al.* Harmonics of circadian gene transcription in mammals. PLoS Genet 5, e1000442 (2009). 19343201 10.1371/journal.pgen.1000442 PMC2654964

[b5] Fu L. & Lee C. C. The circadian clock: pacemaker and tumour suppressor. Nat Rev Cancer 3, 350–361 (2003). 12724733 10.1038/nrc1072

[b6] Yang X. *et al.* Nuclear receptor expression links the circadian clock to metabolism. Cell 126, 801–810 (2006). 16923398 10.1016/j.cell.2006.06.050

[b7] Keller M. *et al.* A circadian clock in macrophages controls inflammatory immune responses. Proc Natl Acad Sci USA 106, 21407–21412 (2009). 19955445 10.1073/pnas.0906361106 PMC2795539

[b8] Levi F. & Schibler U. Circadian rhythms: mechanisms and therapeutic implications. Annu. Rev. Pharmacol. Toxicol. 47, 593–628 (2007). 17209800 10.1146/annurev.pharmtox.47.120505.105208

[b9] Yan J., Wang H., Liu Y. & Shao C. Analysis of gene regulatory networks in the mammalian circadian rhythm. PLoS Comp Biol 4, e1000193 (2008). 10.1371/journal.pcbi.1000193 PMC2543109 18846204

[b10] Storch K.-F. *et al.* Extensive and divergent circadian gene expression in liver and heart. Nature 417, 78–83 (2002). 11967526 10.1038/nature744

[b11] Košir R. *et al.* Determination of reference genes for circadian studies in different tissues and mouse strains. BMC Mol Biol 11, 60 (2010). 20712867 10.1186/1471-2199-11-60 PMC2928770

[b12] Oster H., Damerow S., Hut R. A. & Eichele G. Transcriptional profiling in the adrenal gland reveals circadian regulation of hormone biosynthesis genes and nucleosome assembly genes. J Biol Rhythms 21, 350–361 (2006). 16998155 10.1177/0748730406293053

[b13] Forger D. B. & Peskin C. S. A detailed predictive model of the mammalian circadian clock. Proc Natl Acad Sci USA 100, 14806–14811 (2003). 14657377 10.1073/pnas.2036281100 PMC299807

[b14] Mirsky H. P., Liu A. C., Welsh D. K., Kay S. A. & Doyle F. J. A model of the cell-autonomous mammalian circadian clock. Proc Natl Acad Sci USA 106, 11107–11112 (2009). 19549830 10.1073/pnas.0904837106 PMC2699375

[b15] Relogio A. *et al.* Tuning the mammalian circadian clock: robust synergy of two loops. PLoS Comp Biol 7, e1002309 (2011). 10.1371/journal.pcbi.1002309 PMC3240597 22194677

[b16] Korenčič A. *et al.* The interplay of cis-regulatory elements rules circadian rhythms in mouse liver. PLoS One 7, e46835 (2012). 23144788 10.1371/journal.pone.0046835 PMC3489864

[b17] Bintu L. *et al.* Transcriptional regulation by the numbers: Models. Curr Opin Genet Dev 15, 116–124 (2005). 15797194 10.1016/j.gde.2005.02.007 PMC3482385

[b18] Sharova L. V. *et al.* Database for mRNA half-life of 19 977 genes obtained by DNA microarray analysis of pluripotent and differentiating mouse embryonic stem cells. DNA Res 16, 45–58 (2009). 19001483 10.1093/dnares/dsn030 PMC2644350

[b19] Friedel C. C., Doelken L., Ruzsics Z., Koszinowski U. H. & Zimmer R. Conserved principles of mammalian transcriptional regulation revealed by RNA half-life. Nucleic Acids Res 37, e115 (2009). 19561200 10.1093/nar/gkp542 PMC2761256

[b20] Suter D. M. *et al.* Mammalian genes are transcribed with widely different bursting kinetics. Science 332, 472–474 (2011). 21415320 10.1126/science.1198817

[b21] Lee C., Etchegaray J. P., Cagampang F. R., Loudon A. S. & Reppert S. M. Posttranslational mechanisms regulate the mammalian circadian clock. Cell 107, 855–867 (2001). 11779462 10.1016/s0092-8674(01)00610-9

[b22] Preitner N. *et al.* The orphan nuclear receptor REV-ERB controls circadian transcription within the positive limb of the mammalian circadian oscillator. Cell 110, 251–260 (2002). 12150932 10.1016/s0092-8674(02)00825-5

[b23] Hamilton E. E. & Kay S. A. Snapshot: circadian clock proteins. Cell 135, 368 (2008). 18957209 10.1016/j.cell.2008.09.042

[b24] Rey G. *et al.* Genome-wide and phase-specific DNA-binding rhythms of BMAL1 control circadian output functions in mouse liver. PLoS Biol 9, e1000595 (2011). 21364973 10.1371/journal.pbio.1000595 PMC3043000

[b25] Vanselow K. *et al.* Differential effects of PER2 phosphorylation: molecular basis for the human familial advanced sleep phase syndrome (FASPS). Genes Dev 20, 2660–2672 (2006). 16983144 10.1101/gad.397006 PMC1578693

[b26] Košir R. *et al.* Circadian expression of steroidogenic cytochromes P450 in the mouse adrenal gland - involvement of cAMP-responsive element modulator in epigenetic regulation of Cyp17a1. FEBS J. 279, 1584–93 (2012). 21883931 10.1111/j.1742-4658.2011.08317.x

[b27] Meireles-Filho A. C. A., Bardet A. F., Yanez-Cuna J. O., Stampfel G. & Stark A. cis-regulatory requirements for tissue-specific programs of the circadian clock. Curr Biol 24, 110 (2014). 10.1016/j.cub.2013.11.017 24332542

[b28] Hansen A. S. & O'Shea E. K. Promoter decoding of transcription factor dynamics involves a trade-off between noise and control of gene expression. Mol Syst Biol 9, 704 (2013). 24189399 10.1038/msb.2013.56 PMC4039373

[b29] Koike N. *et al.* Transcriptional architecture and chromatin landscape of the core circadian clock in mammals. Science 338, 349–354 (2012). 22936566 10.1126/science.1226339 PMC3694775

[b30] Bugge A. *et al.* Rev-erb*α* and Rev-erb*β* coordinately protect the circadian clock and normal metabolic function. Genes Dev 26, 657–667 (2012). 22474260 10.1101/gad.186858.112 PMC3323877

[b31] Feng D. *et al.* A circadian rhythm orchestrated by histone deacetylase 3 controls hepatic lipid metabolism. Science 331, 1315–1319 (2011). 21393543 10.1126/science.1198125 PMC3389392

[b32] Cho H. *et al.* Regulation of circadian behaviour and metabolism by REV-ERB-*α* and REV-ERB-*β*. Nature 485, 123–127 (2012). 22460952 10.1038/nature11048 PMC3367514

[b33] Gery S. *et al.* Transcription profiling of C/EBP targets identifies Per2 as a gene implicated in myeloid leukemia. Blood 106, 2827–2836 (2005). 15985538 10.1182/blood-2005-01-0358 PMC1895299

[b34] Reinke H. *et al.* Differential display of DNA-binding proteins reveals heat-shock factor 1 as a circadian transcription factor. Genes Dev 22, 331–345 (2008). 18245447 10.1101/gad.453808 PMC2216693

[b35] Gerber A. *et al.* Blood-borne circadian signal stimulates daily oscillations in actin dynamics and SRF activity. Cell 152, 492–503 (2013). 23374345 10.1016/j.cell.2012.12.027

[b36] Kojima S., Sher-Chen E. L. & Green C. B. Circadian control of mRNA polyadenylation dynamics regulates rhythmic protein expression. Genes Dev 26, 2724–2736 (2012). 23249735 10.1101/gad.208306.112 PMC3533077

[b37] Jouffe C. *et al.* The circadian clock coordinates ribosome biogenesis. PLoS Biol 11, e1001455 (2013). 23300384 10.1371/journal.pbio.1001455 PMC3536797

[b38] Westermark P. O. & Herzel H. Mechanism for 12 hr rhythm generation by the circadian clock. Cell Rep 3, 1228–1238 (2013). 23583178 10.1016/j.celrep.2013.03.013

[b39] Ukai-Tadenuma M. *et al.* Delay in feedback repression by Cryptochrome 1 is required for circadian clock function. Cell 144, 268–281 (2011). 21236481 10.1016/j.cell.2010.12.019

[b40] Oishi K. *et al.* Genome-wide expression analysis of mouse liver reveals clock-regulated circadian output genes. J Biol Chem 278, 41519–41527 (2003). 12865428 10.1074/jbc.M304564200

[b41] Bozek K. *et al.* Regulation of clock-controlled genes in mammals. PLoS One 4, e4882 (2009). 19287494 10.1371/journal.pone.0004882 PMC2654074

[b42] Huang D. W., Sherman B. T. & Lempicki R. A. Systematic and integrative analysis of large gene lists using DAVID bioinformatics resources. Nat Protoc 4, 44–57 (2009). 19131956 10.1038/nprot.2008.211

[b43] Ohsugi M. *et al.* Glucose and insulin treatment of insulinoma cells results in transcriptional regulation of a common set of genes. Diabetes 53, 1496–1508 (2004). 15161754 10.2337/diabetes.53.6.1496

[b44] Luo X., Ikeda Y. & Parker K. L. A cell-specific nuclear receptor is essential for adrenal and gonadal development and sexual dierentiation. Cell 77, 481–490 (1994). 8187173 10.1016/0092-8674(94)90211-9

[b45] Mora S. & Pessin J. E. The MEF2A isoform is required for striated muscle-specific expression of the insulin-responsive GLUT4 glucose transporter. J Biol Chem 275, 16323–16328 (2000). 10748204 10.1074/jbc.M910259199

[b46] Pikkarainen S., Tokola H., Kerkela R. & Ruskoaho H. GATA transcription factors in the developing and adult heart. Cardiovasc Res 63, 196–207 (2004). 15249177 10.1016/j.cardiores.2004.03.025

[b47] Hai T. & Hartman M. G. The molecular biology and nomenclature of the activating transcription factor/cAMP responsive element binding family of transcription factors: activating transcription factor proteins and homeostasis. Gene 273, 111 (2001). 10.1016/s0378-1119(01)00551-0 11483355

[b48] Oishi K. *et al.* Genome-wide expression analysis reveals 100 adrenal gland-dependent circadian genes in the mouse liver. DNA Res 12, 191–202 (2005). 16303750 10.1093/dnares/dsi003

[b49] Menet J. S., Rodriguez J., Abruzzi K. C. & Rosbash M. Nascent-seq reveals novel features of mouse circadian transcriptional regulation. eLife 1, e00011 (2012). 23150795 10.7554/eLife.00011 PMC3492862

[b50] Schwanhausser B. *et al.* Global quantification of mammalian gene expression control. Nature 473, 337–342 (2011). 21593866 10.1038/nature10098

[b51] Robles M. S., Cox J. & Mann M. In-vivo quantitative proteomics reveals a key contribution of post-transcriptional mechanisms to the circadian regulation of liver metabolism. PLoS Genet 10, e1004047 (2014). 24391516 10.1371/journal.pgen.1004047 PMC3879213

[b52] Mauvoisin D. *et al.* Circadian clock-dependent and -independent rhythmic proteomes implement distinct diurnal functions in mouse liver. Proc Natl Acad Sci USA 111, 167–172 (2014). 24344304 10.1073/pnas.1314066111 PMC3890886

[b53] Ouyang Z., Zhou Q. & Wong W. H. Chip-seq of transcription factors predicts absolute and differential gene expression in embryonic stem cells. Proc Natl Acad Sci USA 106, 21521–21526 (2009). 19995984 10.1073/pnas.0904863106 PMC2789751

