# Supplementary material for: Timing of circadian genes in mammalian tissues
Source: Sci Rep. 2014 Jul 22;4:5782. doi: 10.1038/srep05782 (PMC5376044; doi:10.1038/srep05782)
Supplement: Supplementary Information [file srep05782-s1.pdf]

Supplementary information for
Timing of circadian genes in mammalian tissues

18th June 2014

Anja Korenčič, Rok Košir, Grigory Bordyugov, Robert Lehmann, Damjana Rozman, Hanspeter Herzel

Supplementary figures

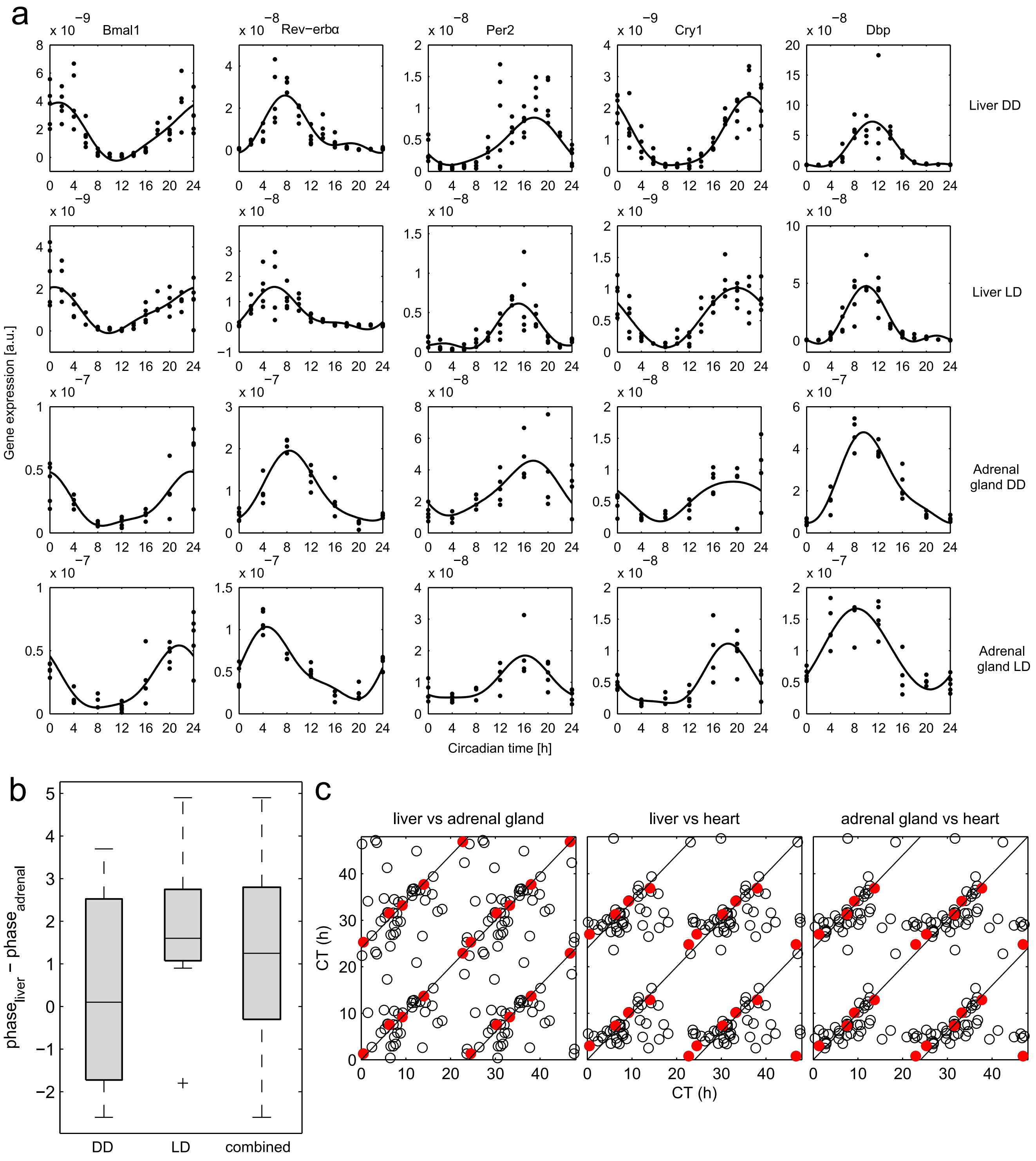

Supplementary Figure S1: **qPCR data.** **a** - Circadian expression of 5 core clock genes in liver and adrenal gland and regression using Equation S1. **b** - Phase difference in gene expression of 8 clock genes in the liver and adrenal gland. **c** - Comparison of phases of genes that are circadian in the liver, adrenal gland, and heart. Core-clock genes *Bmal1*, *Clock*, *Dbp*, *Rev-erbA*, *Per2* are marked in red. The mean phase difference of core clock genes is 0.8 ± 0.43 hours whereas the mean phase difference between other common circadian genes is 3.13 ± 2.01 hours. This confirms the expectation that the core clock is less variable.

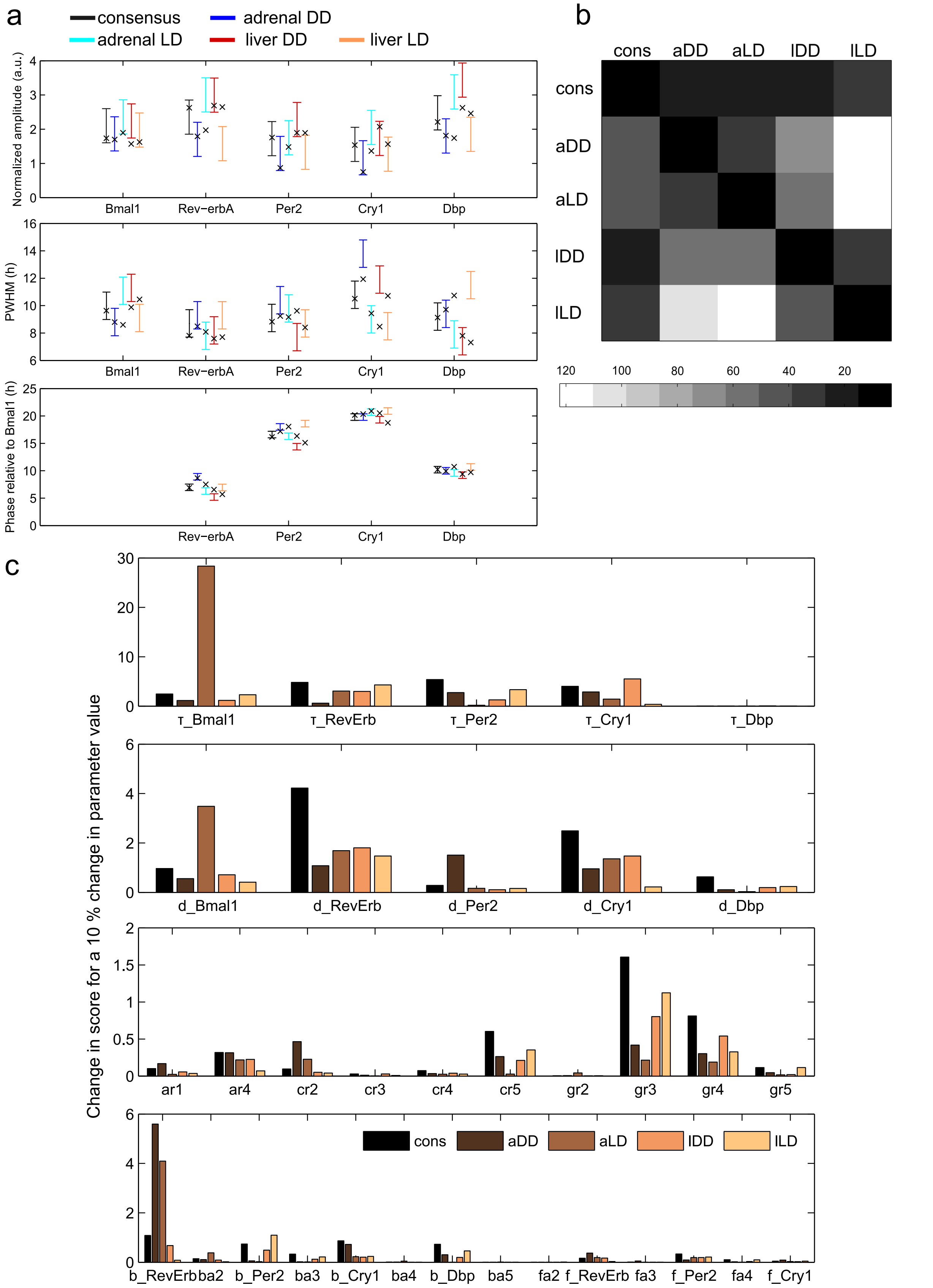

Supplementary Figure S2: **Characterization of the final fits.** **a** - Self-assessed tolerance ranges (tol_{phase}, tol_{ampl}, and tol_{pwhm} from Equation S10) for the descriptive parameters are shown as the colored ranges; the mean of the tolerance ranges are the measured descriptive parameter values (exp_{phase}, exp_{ampl}, and exp_{pwhm}). Crosses show the final values of the fitted model (sim_{phase}, sim_{ampl}, and sim_{pwhm}) for adrenal gland and liver in DD and LD. **b** - Heat map of scores for different combinations of parameter sets and target exp_{period}, exp_{phase}, exp_{ampl}, and exp_{pwhm} values. **c** - Effects (average fold change) of 10 % parameter variations on score for different conditions - control analysis. Explicit delays and degradation rates have the strongest effect.

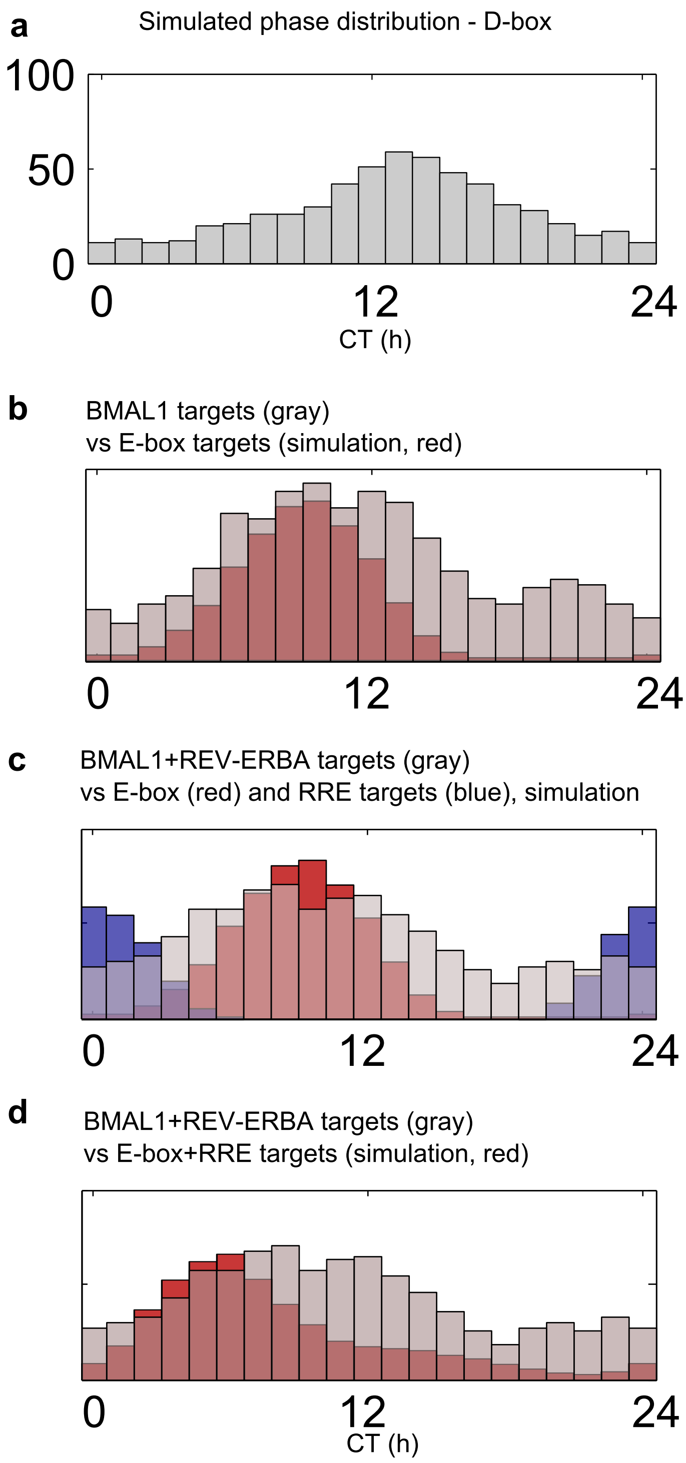

Supplementary Figure S3: **a - Simulated phase distribution for output genes with D-boxes**, related to Fig. 3a. Peak expression of D-box targets can be observed at around CT 14. **b-d - Comparison of experimental and simulated peak phase distributions.** **b** - E-box targets have a peak around CT 10 (as predicted by simulations) but many other phases as well. This points to additional regulators as discussed extensively in the main text. **c** - A simple superposition of E-box simulations (red) and RRE simulations (blue) can be compared with peak phase distribution from genes that are BMAL1 and REV-ERBA targets (grey). Obviously, the experimental data for E-box and RRE-targets are quite different. **d** - If we simulate the combinatorial and multiplicative effects of E-box and RREs (red) and compare them to peak phase distribution from genes that are BMAL1 and REV-ERBA targets (gray), we obtain a better agreement with experimental data. In particular, the RRE-elements shift the distribution to earlier phases (compared to pure E-boxes) and broaden the distribution. Still, there are clear deviations pointing to co-regulators and post-transcriptional effects.

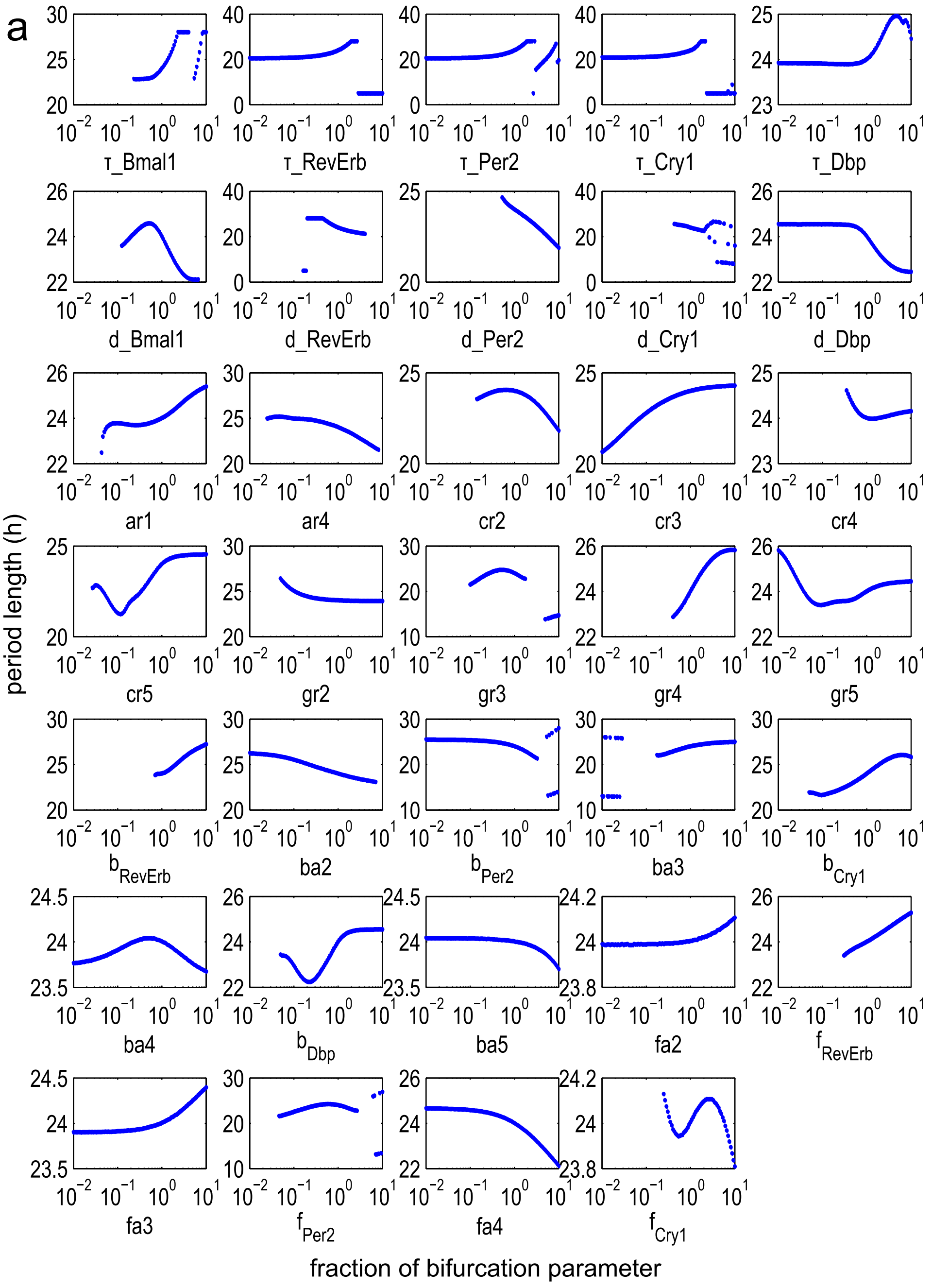

Supplementary Figure S4: **Graphical control analysis of the consensus model**. **a** - Effects of parameters on period length. **b** - Effects of parameters on amplitudes. **c** - Effects of parameters on phases. No oscillations are observed in the areas without plotted values.

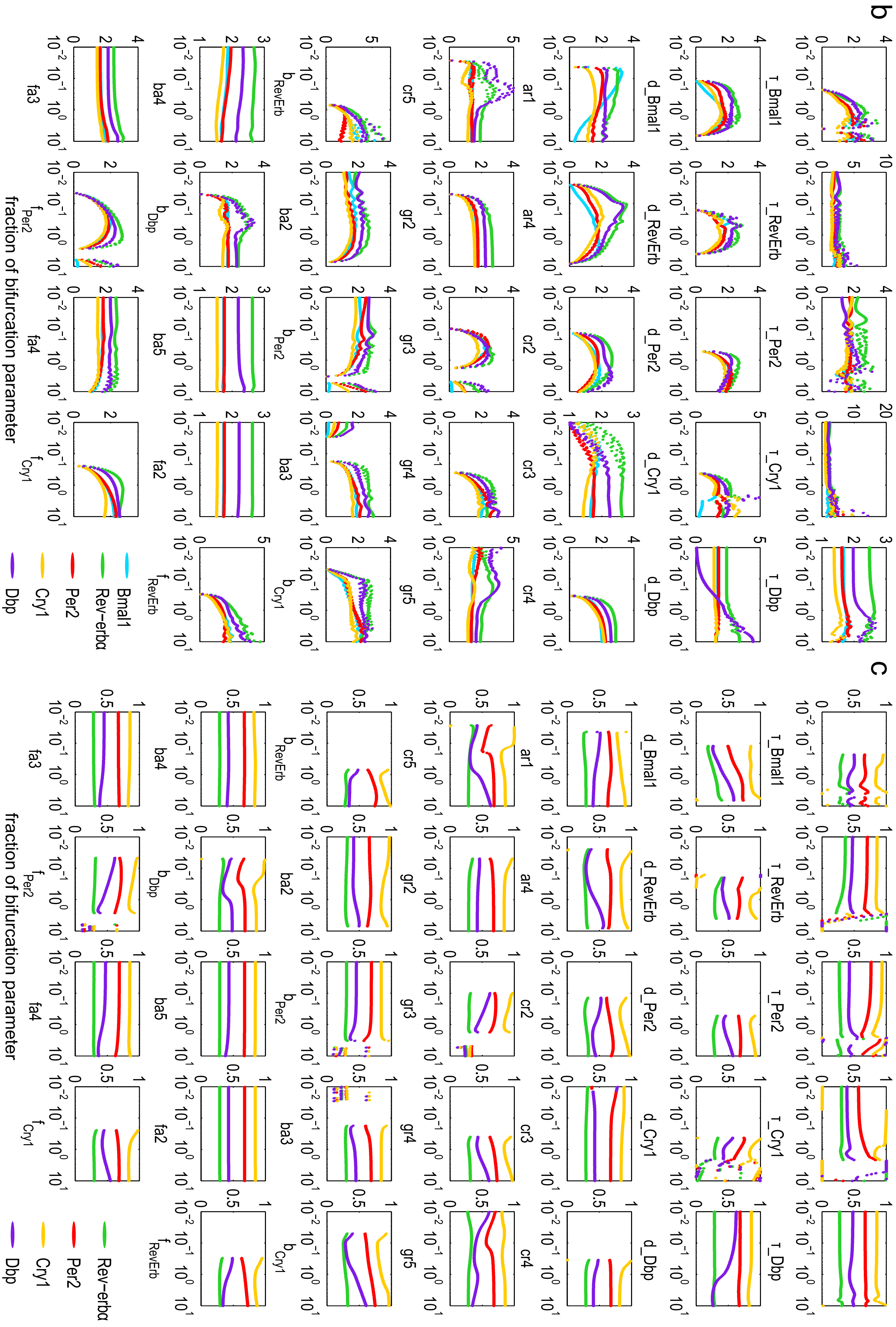
b
τ_Bmal1
τ_RevErb
τ_Per2
τ_Cry1
τ_Dbp
d_Bmal1
d_RevErb
d_Per2
d_Cry1
d_Dbp
ar1
ar4
cr2
cr3
cr4
cr5
gr2
gr3
gr4
gr5
b_{RevErb}
ba2
b_{Per2}
ba3
b_{Cry1}
ba4
b_{Dbp}
ba5
fa2
f_{RevErb}
fa3
f_{Per2}
fa4
f_{Cry1}
fraction of bifurcation parameter
Bmal1
Rev−erbα
Per2
Cry1
Dbp
c
fraction of bifurcation parameter
Rev−erbα
Per2
Cry1
Dbp

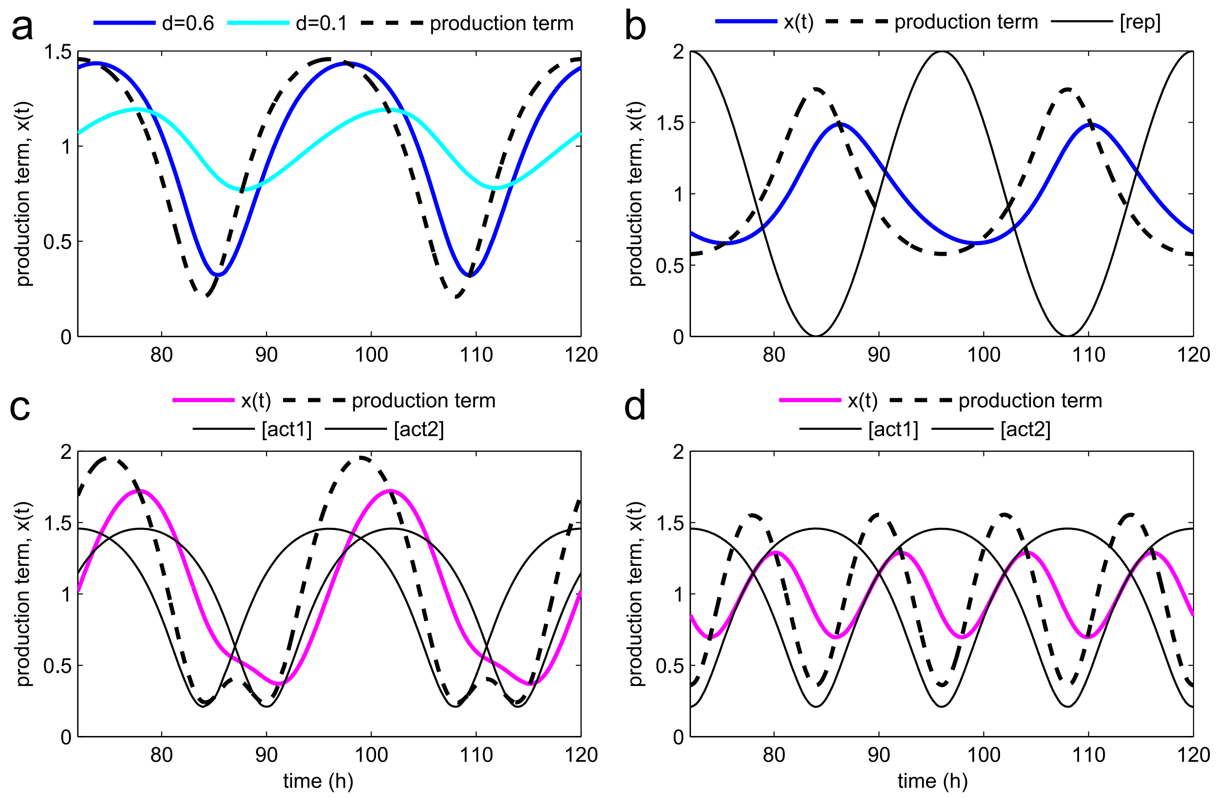

Supplementary Figure S5: **The role of the degradation rate an multiple regulators in determining the phase and amplitude of gene expression. a - single activator.** The solution of Equation S11 for the production term from Equation S15 is plotted. For smaller degradation rates, the gene expression is delayed and the relative amplitude is decreased. **b - single repressor.** The solution of Equation S11 for the production term from Equation S16. Note the out-of-phase oscillations of repressor and target gene. **c - Two activators lead to an intermediate phase.** The solution of Equation S11 for the production term from Equation S17. For two activators with the same amplitude, the phase of the production term is between the peaks of the activators. The degradation step delays the actual mRNA expression compared to the production term itself. **d - Two out-of-phase activators.** The solution of Equation S11 for the production term from Equation S17. For two out-of-phase activators with the same amplitude, a perfect harmonic oscillation with a 12 h period length can be observed. In all cases, $A_{act} = A_{rep} = 1$; $g_{act} = 10$; $K_{act} = K_{rep} = 1$.

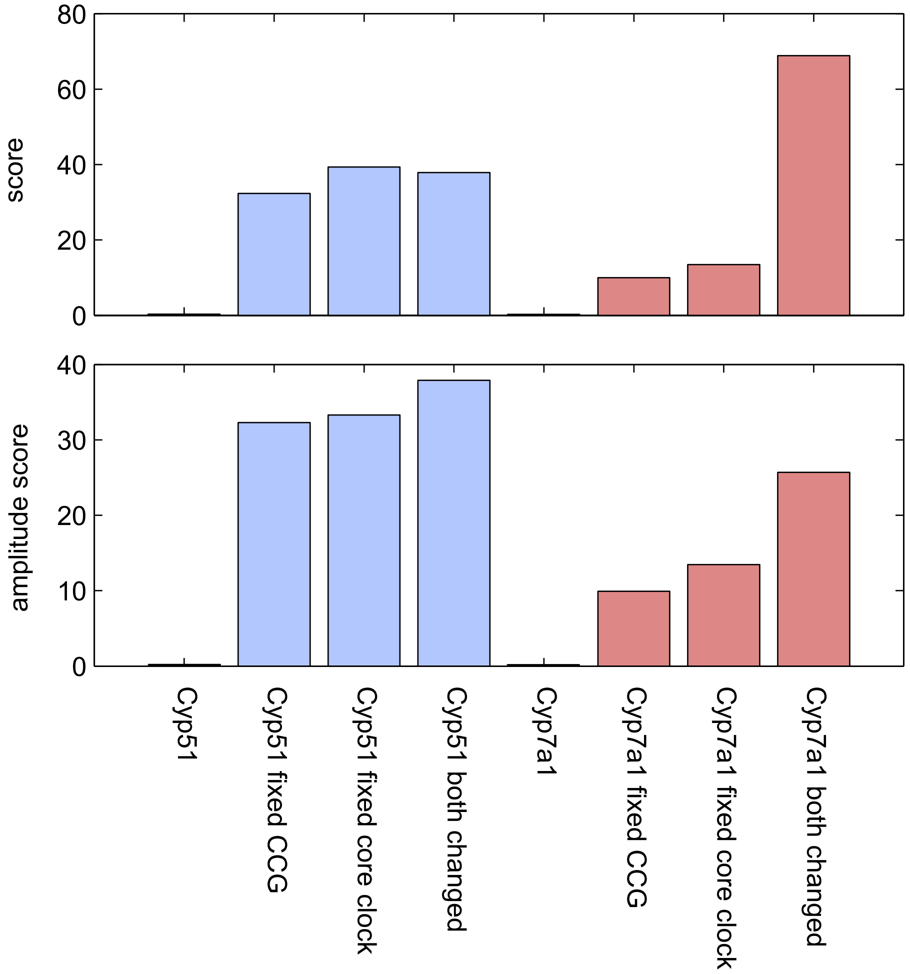

Supplementary Figure S6: **The role of core-clock- and CCG-specific parameters in determining phases and amplitudes of *Cyp* genes.** Above: If core-clock and CCG parameters (including a_{CCG} and d_{CCG}) are optimized, both resulting scores are below 0.02 (left-most columns). Fixing CCG parameters and changing core-clock-parameters (liver DD to adrenal DD) leads to a score of about 32. Varying CCG parameters only leads to similarly high scores. Below: Most of the overall score change comes from the change in the amplitudes of *Cyp* genes.

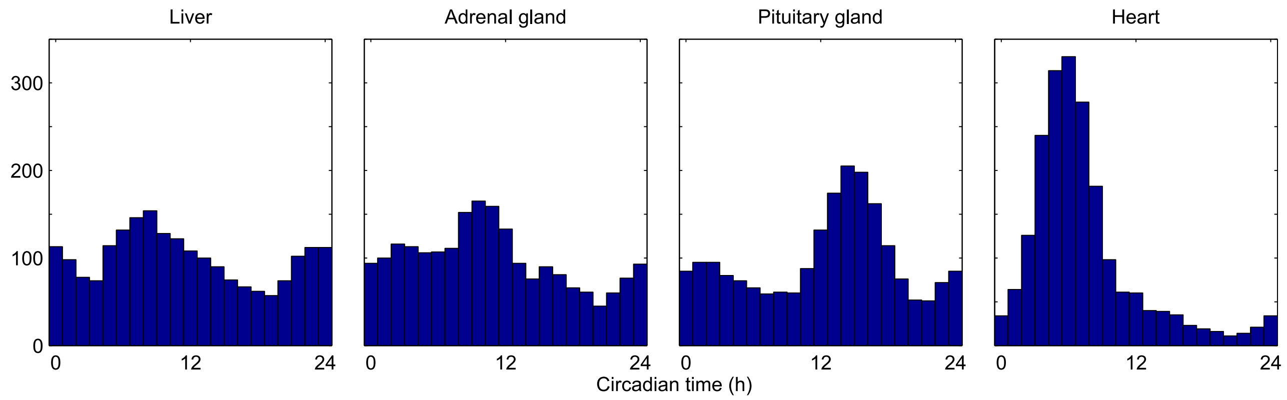

Supplementary Figure S7: **Peak phase distributions of top 500 circadian genes in liver, adrenal gland, pituitary gland, and heart.** There are clear differences between peak phase distributions from all tissues. Especially striking is the difference to peak phase distribution in heart even though the peak phases of core clock genes are similar to other tissues (Fig. 6). Datasets from [1, 2, 3] were used to re-fit the data using our biharmonic fit for liver, adrenal gland and heart. For pituitary gland we display the phases according to the analysis in [1].

Supplementary tables

Supplementary Table S1: **Peak characterisation of five model genes in the liver and adrenal gland for DD and LD.**

	liver DD				liver LD			
	**Phase (CT)**	**Max (a.u.)**	**Min (a.u.)**	**PWHM (h)**	**Phase (CT)**	**Max (a.u.)**	**Min (a.u.)**	**PWHM (h)**
Bmal1	1.4	2.2	-0.1	10.8	0.7	2.1	-0.1	11.4
Rev-erbα	7.7	2.9	-0.1	7.9	5.9	2.8	-0.2	8.3
Per2	17.7	2.0	0.2	9.6	15.1	2.5	0.2	7.5
Cry1	22.1	2.2	0.2	9.0	20.0	1.9	0.1	11.7
Dbp	11.0	3.0	-0.1	7.9	9.9	3.2	-0.2	7.4

	adrenal gland DD				adrenal gland LD			
	**Phase (CT)**	**Max (a.u.)**	**Min (a.u.)**	**PWHM (h)**	**Phase (CT)**	**Max (a.u.)**	**Min (a.u.)**	**PWHM (h)**
Bmal1	23.1	2.1	0.3	8.8	21.6	2.2	0.2	9
Rev-erbα	8.8	2.0	0.3	9.3	4.6	1.9	0.3	9.3
Per2	16.7	1.7	0.4	10.5	16.2	1.8	0.5	8.5
Cry1	19.3	1.5	0.3	13.8	18.5	2.1	0.3	8.4
Dbp	10.2	2.1	0.2	9.5	8.3	1.7	0.4	12.2

Each gene expression profile is characterized by the phase, minimum and maximum (relative amplitude), and peak width at half of the maximum (PWHM).

Supplementary Table S2: **Literature data for some model parameters for the consensus model.**

**Symbol**	**Value (cons)**	**Unit**	**Literature data**
τ_{Bmal1}	4.76	h	Delay of *Bmal1*. Reported values are between 0 h [4] and 8 h [5]. [6] and [7] show that BMAL1 binding has a peak around CT 6, which would suggest a value of about 4 h.
$\tau_{Rev-erb\alpha}$	1.79	h	Delay of *Rev-erb*α. A value of approximately 2 h was reported in [5]. Taking into account ChIP-seq data of [8], the maximal binding of REV-ERBα occurs at ZT 1-5, which would suggest delays between 0 and 4 h.
τ_{Per2}	3.82	h	Delay of *Per2*. [4] report a value of about 6 h. The study of [7] found maximal binding of PER2 to regulatory regions at around CT 17, which suggests shorter delays of 3-4 h.
τ_{Cry1}	3.13	h	Delay of *Cry1*. [4] report a value of about 6 h. Shorter delays are suggested by the study of [7], where maximal binding of CRY1 to regulatory regions occurs at around CT 0, implying delays of 3-4 h.
τ_{Dbp}	2.08	h	Delay of *Dbp*. Range of 0 - 2 h was reported in [9].
d_{Bmal1}	0.40	h^{-1}	Degradation rate of *Bmal1* mRNA. The experimentally determined range is 0.17 - 0.60 h^{-1} [10, 11, 12].
$d_{Rev-erb\alpha}$	0.67	h^{-1}	Degradation rate of *Rev-erb*α mRNA. The experimentally determined range is 0.19 - 0.29 h^{-1} [10, 11].
d_{Per2}	0.51	h^{-1}	Degradation rate of *Per2* mRNA. The experimentally determined range is 0.24 - 0.80 h^{-1} [10, 11].
d_{Cry1}	0.20	h^{-1}	Degradation rate of *Cry1* mRNA. The experimentally determined range: 0.16 - 0.18 h^{-1} [10, 11].
d_{Dbp}	0.56	h^{-1}	Degradation rate of *Dbp* mRNA. The experimentally determined range is 0.18 - 0.36 h^{-1} [10, 11, 12].

Values are shown for the consensus model (other parameter values are shown in Supplementary Dataset 1).

Supplementary datasets

Supplementary dataset 1: qPCR data and primers. Experimental data for measured genes in the liver and adrenal gland and primer sequences used in the study.

Supplementary dataset 2: Control analysis of the consensus model and parameter values. Changes (in %) of amplitudes, phases, period length and score are shown for a 10 % change of a parameter value.

Supplementary dataset 3: List of circadian transcription factors in the liver and adrenal gland.

Supplementary dataset 4: Gene Ontology analysis of circadian probe sets from liver and adrenal gland.

Supplementary methods

Gene expression analysis and choice of model elements

Harmonic regression of gene expression data

Different sampling intervals require careful estimation of phases *via* harmonic fits. Experimental data were fitted by trigonometric functions with 24 h and 12 h period to represent variable waveforms:

$$x_t = a1\sin\left(\frac{2\pi}{24h}t\right) + a2\cos\left(\frac{2\pi}{24h}t\right) + b1\sin\left(\frac{2\pi}{12h}t\right) + b2\cos\left(\frac{2\pi}{12h}t\right) + c. \tag{S1}$$

To get each gene's circadian parameters, we normalized the data by dividing it by the mean level of expression. Gene expression of each gene was described by peak phase, relative amplitude, and width (peak width in hours for the fit with 12 h harmonics). Raw data of qPCR gene expression are presented in the Supplementary Table 1; differences between tissues are larger than typical statistical errors due to sampling. The same fitting procedure was used for analyzing microarray gene expression data ([2, 1, 3]).

Choice of model components - 5 core clock genes

As in a previous model [13] we decided to limit the number of model components. To model the clock in different tissues and conditions we studied four data sets: gene expression in mouse liver and adrenal gland under DD and LD conditions. Our final model describes time-dependent expression of five genes: *Bmal1*, *Rev-erbα*, *Per2*, *Cry1*, and *Dbp*. *Per1,2* and *3* have similar expression profiles and thus *Per2* serves as a representative of this gene group. The supporting role of *Per1* and *Per3* is included implicitly in enlarged kinetic parameters of *Per2* action. *Per2* and *Cry1* thus serve as representatives of early and late negative E-box regulation and *Bmal1* for positive regulation [14]. We found that pairs of transcriptional activators and repressors (*Ror* and *Rev-erb*, *Dbp* and *E4bp4*) peak at nearly opposite phases. Consequently, we chose only one representative of RRE- and D-box-regulators. Anti-phasic regulators of RREs and D-boxes (E4BP4 for DBP, ROR for REV-ERB) are implicitly represented through the different parameters values of the corresponding production terms as discussed previously (Supplement S3 of [13]). Raw data with fits for the 5 chosen genes are presented in Fig. S1.

The expression profiles of each gene can be characterized by descriptive parameters that were later used to fit the model to the data (Table S1).

Phase difference in gene expression between liver and adrenal gland

The work of Oster et al. [2] shows that the genes from peripheral clocks are delayed relative to the SCN clock, which is in line with our understanding of clock hierarchy. Additionally, the adrenal gland influences other peripheral oscillators through glucocorticoids, which leads to the expectation that the expression of circadian genes from other peripheral oscillators would be delayed compared to the adrenal gland. Interestingly, liver genes were expressed earlier than adrenal genes in the data set of [2].

We plotted phase difference in expression of 8 clock genes circadianly expressed in the liver and adrenal gland from our data set (Fig. S1). For our own data, the expression of liver genes is delayed when compared to the same genes in the adrenal gland, and the difference is larger in LD conditions.

Modeling using a set of delay-differential equations

Our modeling approach is based on the regulatory regions of core clock genes. We defined two types of modulators (based on [15]): activator and repressor modulators. The activating modulator describes the activation action of the two transcriptional activators in our system: *Bmal1* (a part of E-box modulator) and *Dbp* (D-box modulator). It is given by the following equation:

$$act(G, b_G, ba_G) = \frac{1 + b_G \frac{G_{(t-\tau_G)}}{ba_G}}{1 + \frac{G_{(t-\tau_G)}}{ba_G}}. \tag{S2}$$

Normalized concentration of an activator (*Bmal1* or *Dbp*) is described by $\frac{G_{(t-\tau_G)}}{ba_G}$, where $G_{(t-\tau_G)}$ is the the value of the variable G, taken at time $(t - \tau_G)$. Parameter τ_G is the explicit delay of gene G, i.e. the time delay between the peak of mRNA expression and the peak of functional protein in the nucleus. It includes processes such as translation, post-translation modification, complex formation, and nuclear translocation. It can be estimated to a certain extent by the literature data of mRNA and protein expression (Table S2). The parameter b_G ($b_G > 1$) represents the fold activation of a target gene through the action of gene G.

Similarly, the repressor modulator is given by

$$rep(G, a_g) = \frac{1}{1 + \frac{G_{(t-\tau_G)}}{a_G}}. \tag{S3}$$

Here, the gene G stands for the three repressors in our system: *Rev-erbα*, *Per2*, and *Cry1*. As above, $\frac{G_{(t-\tau_G)}}{a_G}$ is the normalized concentration of the repressor, and τ_G is the explicit delay.

Equations

The chosen modeling system can be described with 5 delay-differential equations based on the promoter regions of selected genes (Fig. 1c):

$$\frac{d[Bmal1]_t}{dt} = rep^2(Rev\text{–}erb\alpha,\ ar1) - d_{Bmal1} \cdot [Bmal1]_t; \tag{S4}$$

$$\begin{aligned} \frac{d[Rev\text{–}erb\alpha]_t}{dt} &= act^3(Bmal1,\ b2,\ ba2) \cdot rep^3(Per2,\ cr2) \cdot rep^3(Cry1,\ gr2) \\ &\quad act(Dbp,\ f2,\ fa2) - d_{Rev\text{–}erb\alpha} \cdot [Rev\text{–}erb\alpha]_t; \end{aligned} \tag{S5}$$

$$\begin{aligned} \frac{d[Per2]_t}{dt} &= act^2(Bmal1,\ b3,\ ba3) \cdot rep^2(Per2,\ cr3) \cdot rep^2(Cry1,\ gr3) \\ &\quad act(Dbp,\ f3,\ fa3) - d_{Per2} \cdot [Per2]_t; \end{aligned} \tag{S6}$$

$$\begin{aligned} \frac{d[Cry1]_t}{dt} &= act^2(Bmal1,\ b4,\ ba4) \cdot rep^2(Per2,\ cr4) \cdot rep^2(Cry1,\ gr4) \\ &\quad rep^2(Rev\text{–}erb\alpha,\ ar4) \cdot act(Dbp,\ f4,\ fa4) - d_{Cry1} \cdot [Cry1]_t; \end{aligned} \tag{S7}$$

$$\frac{d[Dbp]_t}{dt} = act^3(Bmal1,\, b5,\, ba5) \cdot rep^3(Per2,\, cr5) \cdot rep^3(Cry1,\, gr5) - d_{Dbp} \cdot [Dbp]_t. \tag{S8}$$

The exponents on the modulators represent the numbers of functional clock controlled elements in each gene's promoter region (number of E-boxes in the case of *Bmal1*, *Per2* and *Cry1*; number of RREs in the case of *Rev-erbα*, and number of D-boxes in the case of *Dbp*).

	**E/E'-box**	**RRE**	**D-box**
**Bmal1**		**2** [5, 16]	
**Rev-erbα**	**3** [5, 17, 6]		**1** [17]
**Per2**	**2** [17, 18, 6]		**1** [17, 19]
**Cry1**	**2** [20, 6]	**2** [20]	**1** [20]
**Dbp**	**3** [21, 22, 6]		

Note that the activating transcription factors RORα and RORγ bind to RREs as well. As shown in the Supplementary Dataset 1, these factors have mRNA expression peak around CT 20. Thus after some delay ROR transcription factors enhance the expression of RRE target genes around CT 0 as well. This effect of additional regulators is implicitly included in our parameters $ar1$ and $ar4$. In the same manner, the D-box acting repressor E4BP4 has nearly the opposite expression phase as the activator DBP and, hence, both regulators can be modeled by a single activation term ('D-box modulator').

Compared to the previous model [13], we simplified the kinetic terms and added *Cry1* repression. We also excluded RORγ, since it is implicitly considered as an out-of-phase activator of ROR-elements. We thus reduced our model to a system with 5 DDEs with 34 parameters. Out of those, 5 are degradation rates that can be quite well approximated by experimental measurements [10, 11, 12] and the 5 delays between peak expression of mRNA and protein. The range for these values can also be taken from publications as discussed below.

Modeling output genes

Expression of core clock genes does not differ much between the liver and adrenal gland (Fig. S1, [23]). However, larger differences in phase distributions and small overlap of circadian genes between tissues have been observed for clock output genes (Fig. 6). In this light, it could be expected that the core clock is only affected weakly by systemic signals while the effect of systemic signals is larger for clock-output genes (through secondary TFs, co-factors, tissue-specific nuclear receptors ...). We show that our modeling framework, based on promoter regions of clock and clock-controlled genes, could explain also the phase and tissue specificity of some clock output genes.

Extension of the model to the additional output gene is described with:

$$\begin{aligned} \frac{d[CCG]_t}{dt} &= act^E(Bmal1,\, b,\, ba) \cdot rep^E(Per2,\, cr) \cdot rep^E(Cry1,\, gr) \\ &\quad rep^{RRE}(Rev\text{-}erb\alpha,\, ar) \cdot act^D(Dbp,\, f,\, fa) - d \cdot [CCG]_t. \end{aligned} \tag{S9}$$

Modeling of the output gene follows the same framework as for the core clock model. *Bmal1*, *Per2* and *Cry1* describe the potential E-box regulation $act^{E}(Bmal1,\ b,\ ba) \cdot rep^{E}(Per2,\ cr) \cdot rep^{E}(Cry1,\ gr)$; the exponent E stands for the number of E-boxes. RRE regulation is effective through *Rev-erbα* $rep^{RRE}(Rev\text{–}erb\alpha,\ ar)$ with RRE representing the number of ROR-elements. Similarly, D-box regulation is described through the action of *Dbp* with a term $act^{D}(Dbp,\ f,\ fa)$.

Fitting

Fitting procedure

To fit the model, we applied evolutionary optimization strategies to estimate unknown parameters. For each parameter, we chose initial value and a range in which the parameter space will be sampled. In the first round, random values of parameters within the ranges were chosen and the system was evaluated (dde23 solver) by the score for each parameter set. For the second round, the best scoring parameter set was taken and the process was repeated with a halved range of possible parameter values for each parameter. In total, 10 such rounds were performed to get the final parameter set.

First, we fitted the consensus model using all 4 data sets. The final parameter set was taken as starting parameter values for fitting the other models. Multiple fittings were carried out and for each model version (liver/adrenal gland, DD/LD), multiple minima of the score were examined through control analysis presented below. Parameter values were rounded to 2-3 digits.

Score and descriptive parameter tolerances

We focused on descriptive parameters for the core clock genes rather than the data points themselves. We described each gene with its amplitude, peak width, and phase relative to the phase of *Bmal1*. The model was fitted based on these descriptive parameters with self-assessed tolerance ranges (explained below). During the fitting procedure, each step was evaluated by computing the score for a specific parameter set (Equation S10):

$$score = \frac{(exp_{period} - sim_{period})^2}{tol^2_{period}} + \sum \frac{(exp_{phase} - sim_{phase})^2}{tol^2_{phase}} + \sum \frac{(exp_{ampl} - sim_{ampl})^2}{tol^2_{ampl}} + \sum \frac{(exp_{pwhm} - sim_{pwhm})^2}{tol^2_{pwhm}}. \tag{S10}$$

The parameters exp_{period} and sim_{period} represented measured and simulated period length, respectively. Terms for phases (exp_{phase}, sim_{phase}), amplitudes (exp_{ampl}, sim_{ampl}), and peak widths are constructed in the same manner (exp_{pwhm}, sim_{pwhm}) for all genes.

An important step was assessing the tolerance ranges, since they determine the contributions of all terms in Equation S10. The tolerance for the period length is strict: $tol_{period} = 0.1\,\mathrm{h}$. Based on our previous work [13] we decided that the phases of the core clock genes should have a narrow tolerance range for fitting ($tol_{phase} = 10.6\,\mathrm{min}$). Thus they contribute more to the score than amplitudes and peak widths.

Amplitudes of the core clock genes vary between 1.3 and 3.5. Some genes (*Bmal1*) have almost constant amplitudes in all 4 conditions, whereas some genes exhibit quite strong differences between conditions (*Dbp*). We chose relatively large amplitude tolerances: $tol_{ampl} = 0.5\,\mathrm{a.u.}$ Thus we cover the largest differences between genes but still the amplitudes have somewhat limited impact.

For a perfect sine wave, width of a peak would be 12 h. For the core clock genes, we can observe sharper peaks (*Rev-erbα*) or even broader peaks (*Cry1*). Also in the peak width category, the tolerance range is quite broad: $tol_{pwhm} = 1\,\mathrm{h}$. By using this framework, we can differentiate between really sharp peaks (7-8 h) and really flat peaks (12-13 h).

The self-assessed tolerance ranges with target values exp_{phase}, exp_{ampl}, and exp_{pwhm} are shown in Fig. S2 together with optimized simulated values sim_{phase}, sim_{ampl}, and sim_{pwhm}.

Parameter estimation - literature data

Since the basis of our model is the composition of each gene's regulatory region, the determination of the number of clock-controlled elements (CCE) is crucial. When possible, we took experimentally validated CCEs as in [13]. Numbers of CCEs and citations are shown in section Equations in Supplementary Methods.

To get experimentally determined ranges for some other parameters, published data were investigated. For explicit delays τ we took the time difference between peak of mRNA and peak of protein expression [5, 4, 9] - these values were taken into account to get the range of parameter values in the fitting procedure. Similarly, we determined a range of values for degradation rates from [10, 11, 12]. Deviations of our fitted parameter values from the measured values are smaller than differences between different cell types and different methods.

Transcriptional regulation of clock genes involves many co-factors such as CBP/P300 and numerous epigenetic regulations [7]. For parameters describing transcriptional activation and repression almost no direct measurements are available. Thus kinetic parameters of transcriptional activation and repression can only be regarded as effective values. Consequently, it makes sense to estimate those parameters by fitting our model to the measured expression profiles. For the remaining parameters, we started with the parameter values used in our previously published model [13]. Since we had reasonable starting conditions, parameter values could be effectively optimized with a routine described below.

Analysis of the final model

Comparison of optimized fits

Fig. S2 shows the ranges and final values of the model's descriptive parameters. In addition to the period length, amplitudes, phases and peak widths, the levels of genes were considered. Out of the best-scoring parameter sets, we chose the ones that were best representing the measured amounts of mRNA.

We then analyzed the final fits of all model variants (adrenal gland/liver, DD/LD). All final scores were between 3 and 10. We might also ask how a model that was fitted to a given tissue performs for the other tissues. Calculating a score of a model using other data sets quantifies the distances between a given model and other tissues and conditions. We thus used the five final parameter sets and cross-checked the scores for all possible combinations of parameter sets and target values exp_{period}, exp_{phase}, exp_{ampl}, and exp_{pwhm}. Fig. S2 shows that we get the lowest scores when the correct combination of parameter set and target values exp_{period}, exp_{phase}, exp_{ampl}, and exp_{pwhm} is used (diagonal elements). The score increases for all other combinations, in some cases even above 100 (off-diagonal). The consensus model is by design somewhere between adrenal gland and liver target values. Additionally, we can see that the differences between tissues are larger that the differences between DD and LD conditions. For example, using a parameter set, optimized for adrenal gland DD, gives a score above 100 with exp_{period}, exp_{phase}, exp_{ampl}, and exp_{pwhm} from liver DD.

Control analysis

To analyze the behavior of the 5 models for changing parameter values, a simplified control analysis was performed (Supplementary Dataset 4). We look for changes in system variables in response to changes in parameter values. Each model parameter was varied by $\pm 10\,\%$ and the relative changes in score, period length, amplitudes, and phases of all genes relative to *Bmal1* were calculated.

Control analysis of the consensus model revealed that certain parameters are particularly important. Among these essential parameters are delays (τ_{Bmal1}, $\tau_{Rev-erb\alpha}$, τ_{Per2}, τ_{Cry1}), degradation parameters ($d_{Rev-erb\alpha}$, d_{Cry1}), and parameters quantifying transcription *via* E-boxes and RREs ($gr3$, $b_{Rev-erb\alpha}$, b_{Per2}, b_{Cry1}). There are, however, some differences between control analyses of 5 model versions. For example, $d_{Rev-erb\alpha}$ seems to play a bigger role in the adrenal gland LD, and d_{Per2} is the most important degradation rate in adrenal DD. Among the delays, τ_{Bmal1} is much more important in liver DD than in the other models. Some other parameters (such as b_{Per2}, $cr2$, $cr5$, $gr3$, $b_{Rev-erb\alpha}$) show varying influences on the score.

We compare control analysis for all 5 parameter sets (Fig. S2). Effects of parameters on the score are the highest for delays and degradation rates, because they influence practically all amplitudes, phases, and the period length. Parameters with smaller influence on the score (for example $fa2$, $fa3$, $fa4$) still contribute to certain properties of specific genes. Supplementary Fig. 4 graphically shows the effects of changes of parameter values on period length, phases, and amplitudes of individual model genes. Here parameters were varied over 3 orders of magnitude around the default parameter value (10^0). The analysis shows that the model is robust with respect to parameter changes over about one order of magnitude. Large parameter changes lead to bifurcations

which will be analyzed in a forthcoming study.

Phase determination of CCGs - *Cyp* genes

It is difficult to attribute how much core-clock and gene-specific parameters contribute to the phase and amplitude of an output gene. We show that variation of just a couple of output parameters sets the balance between contributions from different promoter elements and their regulators. We can, however, provide some more detailed analysis of *Cyp* phase determination. In Supplementary Figure S6 we compare the score of the optimized system to the scores when only core-clock, only CCG, and all parameters are varied. The results indicate that contributions of core-clock variability and CCG parameters are comparable. For *Cyp7a1*, the combined effect is greater than the sum of the two individual effects. In the lower graph we show the contributions of amplitudes to the scores. We can see that for *Cyp51* most of the contribution comes from the differences in the amplitudes between different conditions.

It was demonstrated in Fig. 2b that the *Cry1* modulator leads to large *Cyp7a1* amplitudes in liver DD. Here we discuss the regulation in some detail. According to our measurements shown in Fig. 1a, *Cry1* mRNA peaks at CT 20 (yellow arrow). The delay between *Cry1* expression and inhibitory actions is between 3 and 4 h according to the model. Consequently, E-box driven transcription of *Cyp7a1* (shown in Fig. 2b) is inhibited around CT 24. This implies that the *Cry1*-driven E-box modulator has a peak at around CT 12 leading to the expression of *Cyp7a1*. According to the control analysis of our model, D-boxes and RREs have only minor effects on *Cyp7a1* transcription. The half-life of *Cyp7a1* leads to an additional shift of the mRNA peak time as discussed previously [13] (section 'Degradation rate and combinatorial action of modulators determine phase and amplitude of target gene expression' of the Supplementary Experimental Procedures). This example shows how phases are determined by the combined action of activators such as BMAL1 and E-box repressors (CRY1 and PER2).

Degradation rate and combinatorial action of modulators determine phase and amplitude of target gene expression

Rhythmic production term

We can describe the mRNA concentration of a clock output gene as

$$\frac{dx}{dt} = p - d \cdot x, \tag{S11}$$

where x is the concentration of mRNA, p the production term, and d the degradation rate of the mRNA. If the transcription factor driving the transcription is expressed in a circadian manner, we can assume periodic production of the mRNA with the production term p

$$p = a + A\cos(\omega t). \tag{S12}$$

Here, a is the basal transcription rate, A the amplitude of the production term, and $\omega = \frac{2\pi}{24\text{h}} \approx 0.26\text{h}^{-1}$ for a circadian period length of 24 h. For this case, we can solve the equation analytically and write the asymptotic solution as

$$x(t) = \frac{a}{d} + \frac{A}{d^2+\omega^2}\left(d\cos(\omega t) + \omega\sin(\omega t)\right). \tag{S13}$$

Analysis of the solution shows that the peak of mRNA can be strongly influenced by the degradation rate. For large degradation rates ($d \gg \omega$), mRNA is nearly in-phase with the production term (cos-term dominates). For small degradation rates, the sin-term dominates which leads to delays up to 6 h. A more detailed analysis of the role of the degradation rate on the phase and amplitude is provided in the supplemental material to [13].

Gene expression is driven by activator and repressor interplay

In a more realistic example, gene expression can be driven by activators and repressors with circadian expression. We can describe the concentration of an activator or a repressor as:

$$[reg] = 1 + A\cos(\frac{2\pi}{24}t), \tag{S14}$$

where A is the relative amplitude of the regulator's oscillation. According to [15], we can describe the production term p of Equation S11 for an example with one activator as

$$p = \frac{1 + g_{act}\frac{[reg]}{K_{reg}}}{1 + \frac{[reg]}{K_{reg}}}, \tag{S15}$$

where g_{act} is the factor with which the activator modifies the gene expression rate and K_{reg} is a scaled dissociation constant for the activator.

For a single repressor, a similar production term can be constructed:

$$p = \frac{1}{1 + \frac{[reg]}{K_{reg}}}. \tag{S16}$$

According to the detailed analysis in [24], interesting behavior arises when two separate circadian transcription factors bind to separate binding sites. For two out-of-phase activators, the production term takes the form of

$$p = \frac{1 + g_1 \frac{1 + A\cos(\frac{2\pi}{24}t)}{K_1}}{1 + \frac{1 + A\cos(\frac{2\pi}{24}t)}{K_1}} \times \frac{1 + g_2 \frac{1 + A\cos(\frac{2\pi}{24}t - \pi)}{K_2}}{1 + \frac{1 + A\cos(\frac{2\pi}{24}t - \pi)}{K_2}}. \tag{S17}$$

Additionally, an activator and repressor can regulate a target gene transcription independently at the same time:

$$p = \frac{1 + g_{act} \frac{1 + A_{act}\cos(\frac{2\pi}{24}t)}{K_{act}}}{1 + \frac{1 + A_{act}\cos(\frac{2\pi}{24}t)}{K_{act}}} \times \frac{1}{1 + \frac{1 + A_{rep}\cos(\frac{2\pi}{24}t)}{K_{rep}}} \tag{S18}$$

The solutions of Equation S11 for the production terms described in Equations S15-S17 are shown in Fig. S5. Solutions for different degradation rates are plotted to show the role of the degradation rate in the phase determination of gene expression.

References

[1] Hughes, M. E. *et al.* Harmonics of circadian gene transcription in mammals. *PLoS Genet* **5**, e1000442 (2009).

[2] Oster, H., Damerow, S., Hut, R. A. & Eichele, G. Transcriptional profiling in the adrenal gland reveals circadian regulation of hormone biosynthesis genes and nucleosome assembly genes. *J Biol Rhythms* **21**, 350–361 (2006).

[3] Storch, K.-F. *et al.* Extensive and divergent circadian gene expression in liver and heart. *Nature* **417**, 78–83 (2002).

[4] Lee, C., Etchegaray, J. P., Cagampang, F. R., Loudon, A. S. & Reppert, S. M. Posttranslational mechanisms regulate the mammalian circadian clock. *Cell* **107**, 855–867 (2001).

[5] Preitner, N. *et al.* The orphan nuclear receptor REV-ERBα controls circadian transcription within the positive limb of the mammalian circadian oscillator. *Cell* **110**, 251–260 (2002).

[6] Rey, G. *et al.* Genome-wide and phase-specific dna-binding rhythms of bmal1 control circadian output functions in mouse liver. *PLoS Biol* **9**, e1000595 (2011).

[7] Koike, N. *et al.* Transcriptional architecture and chromatin landscape of the core circadian clock in mammals. *Science* **338**, 349–354 (2012).

[8] Bugge, A. *et al.* Rev-erba and rev-erbb coordinately protect the circadian clock and normal metabolic function. *Genes Dev* **26**, 657–667 (2012).

[9] Hamilton, E. E. & Kay, S. A. Snapshot: circadian clock proteins. *Cell* **135**, 368 (2008).

[10] Sharova, L. V. *et al.* Database for mRNA half-life of 19 977 genes obtained by DNA microarray analysis of pluripotent and differentiating mouse embryonic stem cells. *DNA Res* **16**, 45–58 (2009).

[11] Friedel, C. C., Doelken, L., Ruzsics, Z., Koszinowski, U. H. & Zimmer, R. Conserved principles of mammalian transcriptional regulation revealed by RNA half-life. *Nucleic Acids Res* **37**, e115 (2009).

[12] Suter, D. M. *et al.* Mammalian genes are transcribed with widely different bursting kinetics. *Science* **332**, 472–474 (2011).

[13] Korenčič, A. *et al.* The interplay of cis-regulatory elements rules circadian rhythms in mouse liver. *PLoS One* **7**, e46835 (2012).

[14] Ko, C. H. & Takahashi, J. S. Molecular components of the mammalian circadian clock. *Hum Mol Genet* **15 Spec No 2**, R271–R277 (2006).

[15] Bintu, L. *et al.* Transcriptional regulation by the numbers: Models. *Curr Opin Genet Dev* **15**, 116–124 (2005).

[16] Yamamoto, T. *et al.* Transcriptional oscillation of canonical clock genes in mouse peripheral tissues. *BMC Mol Biol* **5**, 18 (2004).

[17] Ueda, H. R. *et al.* System-level identification of transcriptional circuits underlying mammalian circadian clocks. *Nat Genet* **37**, 187–192 (2005).

[18] Ogawa, Y. *et al.* Positive autoregulation delays the expression phase of mammalian clock gene per2. *PLoS One* **6**, e18663 (2011).

[19] Yamajuku, D. *et al.* Identification of functional clock-controlled elements involved in differential timing of Per1 and Per2 transcription. *Nucleic Acids Res* **38**, 7964–7973 (2010).

[20] Ukai-Tadenuma, M. *et al.* Delay in feedback repression by Cryptochrome 1 is required for circadian clock function. *Cell* **144**, 268–281 (2011).

[21] Ripperger, J. A. & Schibler, U. Rhythmic CLOCK-BMAL1 binding to multiple E-box motifs drives circadian Dbp transcription and chromatin transitions. *Nat Genet* **38**, 369–374 (2006).

[22] Stratmann, M., Stadler, F., Tamanini, F., van der Horst, G. T. J. & Ripperger, J. A. Flexible phase adjustment of circadian albumin D site-binding protein (dbp) gene expression by cryptochrome1. *Genes Dev* **24**, 1317–1328 (2010).

[23] Oishi, K. *et al.* Genome-wide expression analysis reveals 100 adrenal gland-dependent circadian genes in the mouse liver. *DNA Res* **12**, 191–202 (2005).

[24] Westermark, P. O. & Herzel, H. Mechanism for 12 hr rhythm generation by the circadian clock. *Cell Rep* **3**, 1228–1238 (2013).
